# Supplementary material for: Testing effectiveness and implementation of a standardized approach to sexual dysfunction screening among adolescent and young adult-aged survivors of childhood cancer: A type I hybrid, mixed methods trial protocol
Source: PLoS One. 2024 Jul 22;19(7):e0305677. doi: 10.1371/journal.pone.0305677 (PMC11262696; doi:10.1371/journal.pone.0305677)
Supplement: S1 File — Aim 1 represents a separate intervention adaptation phase. (DOCX) [file pone.0305677.s001.docx]

**Stakeholder-Engaged Development and Evaluation of a Screening Approach for Sexual Dysfunction in Adolescent and Young Adult Patients With and Surviving Childhood Cancer**

| **Protocol Number:** | 22-0709 |
| --- | --- |
| **Principal Investigator:** | Jenna Demedis  13123 E 16^th^ Ave B115  Aurora, CO 80045 |
| **Coordinating Center and Lead Principal Investigator:** | University of Colorado School of Medicine  Jenna Demedis |
| **Funded by:** | NIH NCI |

# LIST OF ABBREVIATIONS

| ASCO | American Society of Clinical Oncology |
| --- | --- |
| AYA | Adolescent and young adult |
| AYA-CCS | Adolescent and young adult-aged childhood cancer survivors |
| CCS | Childhood cancer survivor(s) |
| CFIR | Consolidated Framework for Implementation Research |
| COG | Children’s Oncology Group |
| EHR | Electronic health record |
| NCCN | National Comprehensive Cancer Network |
| PRO | Patient-reported outcome |
| PROMIS | Patient Reported Outcome Measurement Information System |
| SexFS Brief | Patient-Reported Outcomes Measurement Information System Brief Profiles for Sexual Function and Satisfaction |
| RE-AIM | Reach Effectiveness Adoption Implementation and Maintenance |
| SD | Sexual dysfunction |

# PARTICIPATING SITES

Seattle Children’s Hospital

# 1 PROTOCOL SUMMARY

## **1.1 SYnopsis**

| **Protocol Title:** | *Stakeholder-Engaged Development and Evaluation of a Screening Approach for Sexual Dysfunction in Adolescent and Young Adult Patients With and Surviving Childhood Cancer* |
| --- | --- |
| **Objectives:** | ***Overall Objective:*** *The overarching goal of this proposal is to develop and pilot test a patient-centered approach to assessing AYA patients with and surviving childhood cancer (age 15-24) for SD.*  ***ADAPTATION PHASE***   - ***AIM 1***   ***Primary Objective:*** *Iteratively test and refine a routine standardized SD screening and implementation approach in a pediatric oncology clinic using stakeholder input.*  ***Secondary Objective:*** *To develop an implementation package for use of the routine screening approach developed as a primary objective.*  ***HYBRID EFFECTIVENESS-IMPLEMENTATION PHASE***   - ***AIM 2***   ***Primary Objective:*** *Evaluate the preliminary effectiveness of the standardized screening approach for SD (developed in Aim 1) on occurrence of patient-provider SD communication,* *as measured by the rate of patient-reported SD conversations with providers.*  ***Secondary Objective:*** *Evaluate secondary measures of effectiveness, including patient satisfaction, and referral patterns before and after implementation of SD screening.*   - ***AIM 3***   ***Primary Objective:*** *Assess preliminary implementation outcomes (reach, adoption and implementation outcomes of acceptability, appropriateness, feasibility, and fidelity), associated with implementation of the standardized screening approach for SD.*  ***Secondary Objective:*** *Assess contextual factors associated with implementation of the standardized screening approach for SD.* |
|  |  |
| **Endpoint:** | - ***AIM 1***   ***Primary Endpoint:***  *Development of a refined screening approach after 3-5 rounds of iterative testing and modifications, as defined by three stopping criteria:*  *1) Saturation in key informant interviews regarding identified barriers/facilitators (i.e., no new unaddressed barriers/facilitators identified)*  *2) At least 80% of each subgroup (patient, medical) of stakeholders surveyed will have scores of 4 or higher on the adapted AIM, FIM, and IAM (indicating an average response of “agreeing” or “strongly agreeing” to items assessing the acceptability, feasibility, and appropriateness of the screening approach)*  *3) Stakeholders will identify no major problems with the screening approach (note that in the user-centered design field, “major problems” are defined as those which would severely limit the ability of end-users to implement the screen approach).*  ***Secondary Endpoint:*** *Implementation package/materials for use of the routine screening approach developed as a primary objective.*   - ***AIM 2:***   ***Primary Endpoint****: Comparison of pre- and post- results of a patient-reported indicator that his/her provider has communicated with them about SD*  ***Secondary Endpoints:*** *Comparison of pre- and post- results patient satisfaction with SD communication, patient-report that an SD need was met*  ***Exploratory Endpoints:*** *Feasibility of collecting EHR data (incidence of documented/detected SD), number of referrals to SD-related specialty care via EHR order or note documentation*   - ***AIM 3:***   ***Primary Endpoints:*** *Reach (record abstraction evaluating the proportion of eligible patients who completed SD screening); Representativeness (comparison of sociodemographic characteristics between eligible patients who did versus did not receive screening); Adoption:* *medical stakeholder survey score of a 5-point scale on a survey at the conclusion of the study, asking (a) how often they reviewed SD screening results with relevant patients and (b) how often they discussed results with patients; Implementation: Acceptability, appropriateness, feasibility and fidelity will be measured. Medical stakeholder outcomes will be measured at the conclusion of the study using the AIM, IAM and FIM.^2^ Patient-reported outcomes will be measured via 5-point scale questions adapted from the AIM, IAM, and FIM, as well as by a Fidelity Checklist developed in Aim 1.* *The Fidelity Checklist will also be completed through direct study team observation of the screening approach in 10% of patients across sites.*  ***Secondary Endpoints:***  *Reach (secondary): provider response on reasons for missed screening*  *Adoption (secondary):* *results views by the provider (i.e., electronic health record (EHR) clicks)*  *Implementation (secondary): Documented adaptations to the screening approach as they occur throughout the study;* a*t sites where only certain medical stakeholders are responsible for SD screening/follow-up, additional brief interviews assessing acceptability will be completed*  *Contextual Factors: Guided by CFIR and using an explanatory mixed method design, we will complete 10-15 purposive interviews with medical stakeholders who rated the appropriateness, acceptability, or feasibility high or low to better understand contextual factors that serve as barriers or facilitators to use. We will complete an additional 5-10 brief interviews with relevant medical stakeholders who are not directly involved in the intervention.* |
| **Population:** | ***AIM 1***   - ***Patient stakeholders*** *will include with and surviving central nervous system (CNS) cancers, 15-24 years old at the time of study. Detailed inclusion criteria below.* - ***Medical stakeholders*** *will include physicians, nurses and medical assistants in the Children’s Hospital Colorado (CHCO) Neuro-Oncology Program.* - ***Sample size***   *Patient stakeholders*   - - *Maximum number of patient participants that can be enrolled is 35*   - *Minimum number of patient participants to be enrolled is 9)*   *Medical stakeholders*   - - *Maximum number of healthcare provider participants that can be enrolled is 30*   - *Minimum number of healthcare provider participants to be enrolled is 9*   ***AIM 2-3***   - ***Patient stakeholders*** *will include patients with and surviving cancer seen in the CHCO neuro-oncology clinic, CHCO Oncology Clinic, CHCO HOPE Survivorship program, or Seattle Children’s Hospital (SCH) survivorship program, diagnosed prior to age 18 years and 15-24 years old at the time of study.* - ***Medical stakeholders*** *will include physicians, advanced practice provider, and nurses and medical assistants in the CHCO neuro-oncology and oncology clinics, CHCO HOPE Survivorship Program, of the SCH Survivorship Clinic* - ***Sample size***   *Patient stakeholders*   - - *Maximum number of patient participants that can be enrolled is 120*   - *Minimum number of patient participants to be enrolled is 86 (43 pre- and 43 post-implementation of screening approach)*   *Medical stakeholders*   - - *Maximum number of healthcare provider participants that can be enrolled is 50*   - *Minimum number of provider participants to be enrolled is 24 (surveys), 10 (semi-structured interviews), and 5 (brief interviews)* |
| **Study Design:** | *To adhere to NCCN AYA and Survivorship Guidelines, we will be developing and implementing a standardized screening approach for sexual function using the PROMIS SexFS Brief. This intervention will be implemented clinic-wide as standard of care for patients age 15-24 years with or surviving childhood cancer. While the intervention is the use of the screening tool/approach described below, patient responses to the screening measure will not be collected. Separate survey data for the aforementioned endpoints will only be collected for consenting/assenting participants.* |
| **Participating Sites:** | *Children’s Hospital Colorado Anschutz Neuro-Oncology Clinic (Aims 1-3), Children’s Hospital Colorado Oncology Clinic and HOPE Survivorship Program (Aims 2-3), Seattle Children’s Hospital survivorship clinic (Aims 2-3)* |
| **Description of Study Intervention:** | *The screening approach will consist of 1) Standardized use of the PROMIS SexFS Brief in a clinic setting and 2) An implementation package to aid in successful implementation.* |
| **Study Duration:** | *5 years* |
| **Participant Duration:** | *Patient stakeholders: 1 time completion of study surveys within 4 weeks of visit*  *Medical stakeholders:*  *Aim 1: 18 months*  *Aim 2-3: 1 time completion of study surveys and/or qualitative interview within 3 month of study completion* |

## **1.3 STUDY schema**


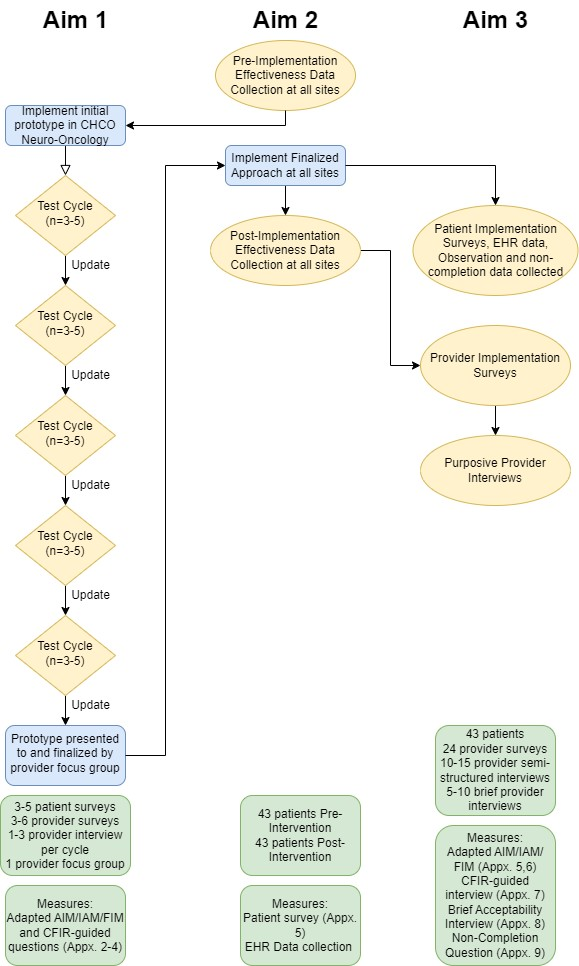


# 2 INTRODUCTION

## **2.1 STUDY RATIONALE**

Our prior research determined adolescent and young adult-aged childhood cancer survivor (AYA-CCS) and medical provider acceptability of the SexFS Brief in a controlled research setting. Development of an acceptable, effective, and feasible screening approach will result in improved recognition of SD in AYA patients with and surviving childhood cancer.

## **2.2 BACKGROUND**

**CCS are at increased risk of SD due to their cancer or treatment history.** SD encompasses lack of desire for sex, arousal difficulties (erection, lubrication), inability to achieve climax/ejaculation, anxiety about sexual performance, climaxing/ejaculating too rapidly, physical pain during intercourse, and lack of pleasure.^3^ SD occurs in 20-50% of CCS, but in clinical practice is widely underrecognized.^4-13^ In CCS, SD may occurs as a result of physiologic and psychosexual reasons, and while certain treatment factors (e.g. cranial or pelvic irradiation, central nervous system tumors, chemotherapy) may exist, demographic, developmental, psychologic and social factors make predicting SD risk difficult. ^4-10,13-22^ Thus, all patients warrant SD screening and education as comprehensive oncology and long-term follow-up care. However, there is no defined standard of care for identification of SD in the AYA childhood cancer population.

**Addressing SD in CCS may result in improved quality of life.** SD in CCS has been associated with poorer quality of life, including bodily pain, general health, social functioning, energy/fatigue, physical quality of life, and mental health.^6-8,13,16,17,21,22^. Importantly, two studies (one of which focused on AYAs) have shown improvement in psychological distress as a result of addressing SD with targeted education and sex-focused therapy.^23,24^ Because SD is common among CCS and is linked to quality of life, evaluating and treating it is critical.

**CCS want improved screening for, and education about, SD after cancer.** In our prior research and in other published work, CCS report that the need to discuss sexuality and cancer is unmet.^13,25-27^ Further, 82% of oncologists and 62% of internists reported rarely discussing sexual function with adult patients who are survivors of adult or pediatric cancer.^28,29^ This low rate of screening contrasts with National Comprehensive Cancer Network and American Society of Clinical Oncology recommendations, which state that the oncology team should initiate regular discussions of sexuality and cancer throughout cancer care and follow-up.^30,31^ Our prior qualitative study, and others, show that AYA patients, both on therapy and in survivorship, would like their providers to initiate conversations to address SD directly, privately and regularly.^26,27,32^ Importantly, many CCS patients report problems with SD but are unaware of the potential relationship between this problem and their health history, further demonstrating the need for provider-initiated conversations.^33^ A clinically feasible, patient-centered approach is necessary to improve screening and treatment for SD among AYA patients with and surviving cancer.

**There are multiple patient- and provider-identified barriers to screening and detection of SD in AYA-CCS.** Pediatric oncologists and internists recognize many challenges in meeting patients’ sexual health needs, including lack of knowledge/experience/training, lack of resources/referrals, parent/family presence, concerns of patient’s or own discomfort, lack of rapport, low priority and limited time.^29,34^ Similarly, AYA-CCS cite barriers including their own discomfort, noticing provider discomfort, presence of family members, and lack of rapport with their providers.^27,32^ The AYA population may be particularly vulnerable to discomfort, both their own and providers’, in discussing SD as a result of developmental stage and concerns about confidentiality; they may therefore be less likely to disclose concerns.^32,35-37^ An approach to screening for SD must address significant patient- and provider-level barriers such as discomfort and privacy, as well as consider setting and system barriers such as limited time, lack of provider experience, and feasibility.

**Use of a screening tool may facilitate SD conversations in the AYA oncology population.** Given the sensitivity of this topic, it is possible that a screening tool may be a helpful approach to assessing for SD both in clinical and research settings.^38^ While no SD screening tools have been specifically validated in the AYA general or CCS population, our prior study demonstrated patient-reported content validity, process validity and acceptability of an existing tool: the National Institute of Health-developed Patient-Reported Outcomes Measurement Information System (PROMIS®) Sexual Function and Satisfaction (SexFS) Brief v2.0 tool (Appendix 1). This tool assesses sexual interest/desire, arousal, discomfort/pain, climax, and satisfaction, among other SD concerns and has comparable sex-specific versions. It has been validated in adult (mean age 58.5 years) cancer populations.^12,39,40^ Further, our ongoing research has demonstrated that use of this tool is viewed favorably by medical stakeholders in the pediatric oncology community. The SexFS Brief is not yet routinely used in clinical practice or research, and there are no published studies evaluating its effectiveness in increasing detection and SD conversations, or implementation outcomes in a clinic setting, or among AYA oncology patients.

**This work has the potential to improve screening and detection of SD in AYA patients with and surviving childhood cancer**, which is the first step to ultimately improving SD and related quality of life outcomes in this population. The American Society of Clinical Oncology published a clinical practice guideline for people with cancer, outlining potential SD concerns and suggested interventions.^31^ Multidisciplinary approaches with medical, education, and therapy components may improve SD and associated distress.^23,24,41^ This study will serve as a necessary first step in improving screening, detection, and treatment of SD in the young AYA oncology population; future work will include establishing the broader effectiveness of our screening approach through a multicenter randomized trial, as well as development, testing, and dissemination of multidisciplinary interventions for SD in this population.

**Preliminary Studies**

We conducted a qualitative study with AYA-CCS (n=24) to describe patient perspectives on SD screening (interim abstract published, manuscript accepted)^27^ and use of the SexFS Brief.^42^ This study demonstrated that while AYA-CCS preferred in-person conversations, they suggested that priming the conversation would increase preparedness/comfort. When asked about ways to provide such a priming, AYA-CCS favored the idea of written information or a questionnaire. Participants suggested that a screening tool would facilitate conversation with their provider by increasing their own comfort and awareness. Participants wanted education and screening to occur regularly throughout cancer therapy and survivorship, but acknowledged that early in treatment they may have lacked the capacity to consider sexual health.^27^ Importantly, this study suggested that the SexFS Brief is a promising tool for SD screening in this population, with patient-reported content validity and acceptability.^42^ Participants preferred a private place to complete the SexFS Brief but were split in preferences regarding setting (in clinic vs. at home) and administration modality (paper vs. electronic). Development of a screening approach using the SexFS Brief in a clinic setting should consider several administration options to promote successful implementation.

In addition to gathering patient stakeholder input in the use of the SexFS Brief, we conducted a (not yet published) qualitative study that has identified medical stakeholder preferences and suggestions for use of the SexFS Brief in a clinical setting and iteratively developed an initial screening prototype via focus groups, integrating provider and AYA-CCS preferences. This study revealed that medical stakeholders find the use of this tool acceptable and feasible and believe that it will improve sexual healthcare of AYA-CCSS. In developing a screening approach, providers valued patient privacy and comfort and emphasized the importance of provider education and patient resource development. Providers also valued the use of electronically delivered screenings that integrated well into the medical record system and standardization in screening across all appropriate patients. Providers were in agreement with AYA-CCS that patients both on and off therapy should receive education and regular screening. The proposed study will iteratively test and modify the developed screening approach in a real clinic setting, using stakeholder feedback. The screening intervention, which is consistent with national guideline recommendations for sexual function evaluation during and off therapy, will be implemented clinic-wide; research measures will be collected only in consenting/assenting participants.

## **2.3 rISK/BENEFIT ASSESSMENT**

### **2.3.1 Known Potential Risks**

There are minimal risks to participants in developing the screening approach. The main risks are breach of confidentiality and privacy; basic demographic and clinical data will be collected, with no identifiable information.

### **2.3.2 Known Potential Benefits**

Participants may receive benefit from study participation if the developed communication techniques between providers and patients leads to improved encounters with healthcare professionals and/or ultimately increases clinical care related to SD concerns.

### **2.3.3 assessment of potential risks and benefits**

The risks to participants are reasonable in relation to the anticipated benefits to participants and/or society, and in relation to the importance of the knowledge that may reasonably be expected to result, thereby falling in favor of performing the study:

- To Participant: Minimal risk, with potential benefit of improved sexual healthcare
- To Society: This is the first study to develop and assess a standardized screening for SD among AYA patients with and surviving childhood cancer. Anticipated outcomes include: 1) a feasible, acceptable, stakeholder-developed SD screening approach; 2) an implementation package to guide future implementation and dissemination at additional clinical sites.
- Justify the importance of the knowledge gained: Improving SD communication and clinical care for AYA oncology patients has the potential to improve quality of life in this vulnerable population. Overall, the study has an excellent risk-benefit ratio.

# 3 OBJECTIVES AND ENDPOINTS

| **Aim** | **OBJECTIVES** | **ENDPOINTS** |
| --- | --- | --- |
| **1** | **Primary:** Iteratively test and refine a routine standardized SD screening and implementation approach in a pediatric oncology clinic using stakeholder input | Development of a refined screening approach after 3-5 rounds of iterative testing and modifications, as defined by three stopping criteria:  1) Saturation in key informant interviews regarding identified barriers/facilitators (i.e., no new unaddressed barriers/facilitators identified)  2) At least 80% of each group of stakeholders (patient, medical) surveyed will have scores of 4 or higher on the AIM, FIM, and IAM (indicating an average response of “agreeing” or “strongly agreeing” to items assessing the acceptability, feasibility, and appropriateness of the screening approach)  3) Stakeholders will identify no major problems with the screening approach (note that in the user-centered design field, “major problems” are defined as those which would severely limit the ability of end-users to implement the screen approach). |
|  | **Secondary:** To develop an implementation package for use of the routine screening approach developed as a primary objective. | Implementation package/materials for use of the routine screening approach developed as a primary objective. |
| **2** | **Primary**: Evaluate the preliminary effectiveness of the standardized screening approach for SD (developed in Aim 1) on occurrence of patient-provider SD communication, as measured by the rate of patient-reported SD conversations with providers. | Comparison of pre- and post- results of a patient-reported indicator that his/her provider has communicated with them about SD |
|  | **Secondary**: Evaluate secondary measures of effectiveness, including patient satisfaction, needs being met | Comparison of pre- and post- results patient satisfaction with SD communication, patient-report that an SD need was met |
|  | **Exploratory/Feasibility:** Feasibility of collecting EHR data to evaluate effectiveness of SD screening intervention | Incidence of documented/detected SD, number of referrals to SD-related specialty care via EHR order or note documentation |
| **3** | **Primary**: Assess preliminary implementation outcomes (reach, adoption and implementation outcomes of acceptability, appropriateness, feasibility, and fidelity), associated with implementation of the standardized screening approach for SD. | Reach: record abstraction evaluating the proportion of eligible patients who completed SD screening  Representativeness: comparison of sociodemographic characteristics between eligible patients who did versus did not receive screening  Adoption: survey score of a 5-point scale on a survey at the conclusion of the study, asking (a) how often they reviewed SD screening results with relevant patients and (b) how often they discussed results with patients; **Implementation**:  Acceptability, appropriateness, feasibility, and fidelity will be measured. Medical stakeholder outcomes will be measured at the conclusion of the study using the AIM, IAM and FIM.2 Patient-reported outcomes will be measured via 5-point scale questions adapted from the AIM, IAM, and FIM, as well as by a Fidelity Checklist developed in Aim 1. The Fidelity Checklist will also be completed through direct study team observation of the screening approach in 10% of patients across sites. |
|  | **Secondary:** Assess contextual factors associated with implementation of the standardized screening approach for SD. | Adoption: results views by the provider (i.e., EHR clicks)  **Implementation**: Fidelity will be evaluated secondarily via documented adaptations to the screening approach as they occur throughout the study  Contextual Factors: Guided by CFIR and using an explanatory mixed method design, we will complete 10-15 purposive interviews with medical stakeholders who rated the appropriateness, acceptability, or feasibility high or low to better understand contextual factors that serve as barriers or facilitators to use. |

# 4 STUDY DESIGN

## **4.1 OVERALL Design**

Aim 1 involves iterative testing and adaptation of the SD screening prototype using stakeholder-engagement and mixed methods. The screening prototype, which has been developed using stakeholder engagement in prior studies, will be implemented across patients as routine clinical care (consistent with national guidelines)^30,31^ in a single clinic (CHCO Neuro-Oncology). There will be 3-5 rounds of iterative testing, stakeholder feedback, and adaptation. This will occur until consensus is reached (described in detail under procedures).

Aims 2 and 3 involve a pilot, type 1, hybrid effectiveness-implementation trial using a pre-post design in 4 clinics. Pre-implementation effectiveness data will be collected via surveys in consenting/assenting patient participants. **Once Aim 1 has been completed, the study protocol will be amended to update intervention details prior to proceeding with implementation across all clinics as routine clinical care.**^30,31^ Following implementation of the screening approach, post-implementation data (effectiveness and implementation outcomes) will be collected via survey and EHR review in consenting/assenting patients. After effectiveness data collection is complete, implementation outcomes will also be assessed via surveys and interviews with consenting provider stakeholders.

*Overview of Project Methods, Participants, Timing and Data/Outcomes*

| **Aim/Years** | **Frameworks, Design**  **and/or Methods** | **Participants** | **Main Data/Outcomes** |
| --- | --- | --- | --- |
| Aim 1: Multistep Iterative SD Screening Approach Development  (Y1-2) | - Frameworks: RE-**AI**M, CFIR - Design: Iterative test/adaptation cycles, explanatory sequential mixed methods - Quantitative: patient and provider surveys - Qualitative: purposive interviewing, focus group | - Neuro-oncology patients ages 15-24 diagnosed with cancer - Neuro-oncology providers (physicians/PA/NP), nurses, and medical assistants (MAs) - Sites: Children’s Hospital Colorado (CHCO) Neuro-Oncology Program | - Finalized screening approach, materials and implementation plan to address barriers/facilitators identified and categorized using CFIR to promote adoption and implementation - Fidelity Checklist to be used in Aim 3 |
| Aim 2: Evaluation of Effectiveness of SD Screening Intervention  Y1 (Pre)  Y3-4 (Post) | - Framework: R**E**-AIM - Design: Type 1 hybrid effectiveness-implementation trial with pre-post design in 3 clinics - Quantitative: Patient surveys, electronic health record, chart review and referral extraction | - Oncology patients ages 15-24 diagnosed with cancer prior to age 18 years - Sites: CHCO HOPE Survivorship Program, CHCO Oncology Clinic, CHCO Neuro-Oncology Program, Seattle Children’s Cancer Survivor Program (SCH) | - Primary outcome: patient-reported SD conversation with a provider - Patient-level secondary outcomes: patient-reporting of SD needs, indication of needs met, patient satisfaction - Provider-level secondary exploratory outcomes: visits with documentation of SD screening, referral patterns |
| Aim 3: Evaluation of Implementation Outcomes and Factors  Y3-4  Y4 (after Aim 2 complete) | - Frameworks: **R**E-**AI**M, CFIR - Design: Type 1 hybrid effectiveness-implementation trial with pre-post design in 3 clinics, explanatory sequential mixed methods - Quantitative: Patient and provider surveys - Qualitative: Purposive qualitative interviews | - Oncology patients ages 15-24 diagnosed with cancer prior to age 18 years - Oncology/survivorship providers (physicians/PA/NP), nurses, and MAs - Sites: CHCO HOPE Survivorship Program, CHCO Oncology Clinic, CHCO Neuro-Oncology Program, Seattle Children’s Cancer Survivor Program (SCH) | - Measurement of reach, fidelity, adoption, acceptability, feasibility, and appropriateness - Exploration of factors affecting implementation to inform future scale-up |

## **4.2 SCIENTIFIC RATIONALE FOR STUDY DESIGN**

The goals of this proposal are to 1) create a feasible and acceptable SD screening approach and 2) develop an implementation package for further implementation and dissemination to effect change for more patients. Implementation science utilizes separate frameworks to evaluate different aspects of a study, including implementation outcomes and contextual factors that influence outcomes (Figure 1).^1,43-45^ This study will use iterative testing and adaptation using stakeholder engagement and mixed methods. The overall structure and outcomes of this proposal are guided by RE-AIM, with a focus on adoption and implementation planning.^46,47^ The Consolidated Framework for Implementation Research (CFIR) will be used in planning and evaluation to understand contextual factors (e.g. facilitators/barriers, in five multilevel domains; see Figure 1) that may influence RE-AIM domains, informing intervention adaptation and future implementation.^48,49^


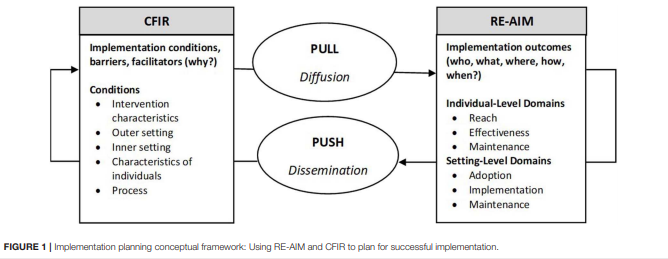


**Implementation conditions, barriers, facilitators (why?)**

**Multilevel Dimensions**

- Intervention characteristics
- Outer setting
- Inner setting
- Characteristics of individuals
- Process

**Implementation outcomes (who, what, where, how, when?)**

**Individual-Level Dimensions**

- Reach
- Effectiveness
- Maintenance

**Setting-Level Dimensions**

- Adoption
- Implementation
- Maintenance

*Figure 2. Implementation planning conceptual framework: using RE-AIM and CFIR to plan for successful implementation (adapted from King 2020).^1^*

Our prior stakeholder-engaged research has resulted in a prototype screening approach that will be implemented across all eligible patients in a single clinic setting to adhere to national guideline recommendations for regular screening for sexual function concerns across the cancer care continuum.^30,31^ We will iteratively adapt our existing stakeholder-informed screening prototype in a real-world clinical setting through cyclical testing and adaptation (Aim 1). Then, we will broaden use of the screening intervention use a type 1, hybrid trial design (Aims 2-3) to simultaneously establish preliminary effectiveness and implementation outcomes of this screening approach. The type 1 hybrid design will facilitate future dissemination by testing the effects of the intervention while simultaneously identifying multilevel barriers and facilitators to real-world implementation.

## **4.3 end of study definition**

Aim 1

Based on recommendations in the literature, we expect that this process will require 3-5 rounds of iterative modifications addressing stakeholder feedback. Three stopping criteria will be used: (1) saturation in key informant interviews regarding identified barriers/facilitators (i.e., no new unaddressed barriers/facilitators identified); (2) at least 80% of each group of stakeholders (patient, medical) surveyed will have scores of 4 or higher on the modified AIM, FIM, and IAM surveys (indicating an average response of “agreeing” or “strongly agreeing” to items assessing the acceptability, feasibility, and appropriateness of the screening approach); and (3) stakeholders will identify no major problems with the screening approach (note that in the user-centered design field, “major problems” are defined as those which would severely limit the ability of end-users to implement the screening approach).

Once these stopping rules are met, the PI, mentors, and collaborators will produce a draft screening approach protocol and implementation package (e.g., use of a “clinical champion”, provider training, use of reminders in the electronic health record (EHR), flexibility for electronic or paper administration). If the stopping rules have not been met after 5 rounds, the research team will decide upon the draft screening approach protocol and implementation package based on feedback elicited through the 5th round. This will be presented for final feedback and modification at a medical stakeholder focus group.

Aim 2-3

Completion of data collection of effectiveness and implementation outcomes for the specified number of patients post-implementation of the sexual function screening tool.

# 5 STUDY POPULATION

## **5.1 patient Inclusion/Exclusion Criteria**

***ADAPTATION PHASE (Aim 1)***

**Inclusion Criteria**

In order to be eligible to participate in this study, an individual must meet all of the following criteria:

1. Provision to sign and date the consent or assent form.
2. Stated willingness to comply with all study procedures and be available for the duration of the study.
3. Be aged 15-24 years old at the time of enrollment
4. Patients with or surviving nervous system (CNS) cancers (must be found in the International Classification of Diseases for Oncology (ICD-O) and have a behavior code ≥2)
5. Must have received cancer-directed therapy with at least one of the following:
   - Chemotherapy: any anticancer drug to treat the cancer diagnosis including immunotherapy
   - Radiotherapy: any radiotherapy to treat the cancer diagnosis
   - Surgery: any surgery to remove cancer including partial or total resections. Biopsies are not considered surgery.
6. Underwent prototype sexual function screening intervention in clinic

**Exclusion Criteria**

An individual who meets any of the following criteria will be excluded from participation in this study:

1. Unable to read and speak English
2. Patients who did not receive cancer-directed therapy
3. Insufficient cognitive functioning to complete study measures, as determined by an oncology provider
4. Patient is at end of life or on hospice, as determined by an oncology provider

***HYBRID EFFECTIVENESS-IMPLEMENTATION PHASE (Aims 2-3)***

**Inclusion Criteria**

In order to be eligible to participate in this study, an individual must meet all of the following criteria:

1. Provision to sign and date the consent or assent form.
2. Stated willingness to comply with all study procedures and be available for the duration of the study.
3. Be aged 15-24 years old at the time of enrollment
4. Patients with or surviving of cancer (must be found in the International Classification of Diseases for Oncology (ICD-O) and have a behavior code ≥2)
5. Must have received cancer-directed therapy with at least one of the following:
   - Chemotherapy: any anticancer drug to treat the cancer diagnosis including immunotherapy
   - Radiotherapy: any radiotherapy to treat the cancer diagnosis
   - Surgery: any surgery to remove cancer including partial or total resections. Biopsies are not considered surgery.
6. Cancer must have been diagnosed before the age of 18 years
7. Patient must have an appointment at one of three clinical sites: CHCO HOPE Survivorship Program, CHCO Oncology Clinic, CHCO Neuro-Oncology Clinic, Seattle Children’s Hospital Survivorship Program

**Exclusion Criteria**

An individual who meets any of the following criteria will be excluded from participation in this study:

1. Unable to read and speak English
2. Patients who did not receive cancer-directed therapy
3. Insufficient cognitive functioning to complete study measures, as determined by an oncology provider Participation in Aim 1 intervention development
4. Patients who did not undergo SD screening within 1 month of being due will be excluded from implementation outcomes (Aim 3)
5. Patient is at end of life or on hospice, as determined by an oncology provider

Consent and assent will consist of paper or REDCap consent/assent with waiver of parental consent and HIPAA waiver for participants <18 years old.

## **5.2 medical stakeholder inclusion/Exclusion Criteria**

***ADAPTATION PHASE (Aim 1)***

**Inclusion Criteria**

In order to be eligible to participate in this study, an individual must meet all of the following criteria:

1. Provision to sign and date the consent form.
2. Stated willingness to comply with all study procedures and be available for the duration of the study.
3. Neuro-oncology providers, including physicians, nurse practitioners, physician assistants, registered nurses (RNs), and medical assistants (MAs) working in the Children's Hospital Colorado neuro-oncology clinic

***HYBRID EFFECTIVENESS-IMPLEMENTATION PHASE (Aim 3)***

**Inclusion Criteria**

In order to be eligible to participate in this study, an individual must meet all of the following criteria:

1. Provision to sign and date the consent form.
2. Stated willingness to comply with all study procedures and be available for the duration of the study.
3. Neuro-oncology, oncology, and survivorship providers, including physicians, nurse practitioners, physician assistants, registered nurses (RNs), and medical assistants (MAs) working at one of the study sites

*Of note, at sites where only certain providers are responsible for SD screening and/or follow-up, the full implementation assessment will be limited to these providers. However, non-responsible providers will also be included for brief acceptability interviews (see study design for details).*

## **5.3 SCREEN FAILURES**

Attempts will be made to screen for eligibility prior to obtaining consent/assent. However, if it is determined after consent/assent has been obtained that the patient is not eligible for any reason, the patient will be removed from the study.

Subject may be re-screened for eligibility with documented approval from the PI.

## **5.4 STRATEGIES FOR RECRUITMENT AND RETENTION**

As previously mentioned, the screening approach will be implemented as part of routine clinical care clinic-wide for patients ages 15-24 with or surviving cancer, adhering to national guidelines regarding sexual health and function clinical care.^30, 31^ Results of the sexual function screening tool will not be collected as part of research.

Research outcomes, as outlined above, will only be collected in consenting/assenting participants.

***ADAPTATION PHASE (AIM 1)***

*Surveys*: **Patient stakeholders** for surveys will be identified by study team; screening will occur through the PI and site PI’s clinical relationships with all patients (see section 10.1.1). Recruitment will occur on a rolling basis during existing clinic visits, or via phone within 4 weeks of the existing appointment, with purposive consecutive sampling to achieve variation across the age categories (age 15-19 and 20-24), patient treatment status (on therapy or survivor), and gender, with at least one member of each of these groups included in each round. Consent and assent will consist of paper or REDCap consent/assent with waiver of parental consent and HIPAA waiver for participants <18 years old (See section 10.1.1.2), presented in person or via email/MyChart/text after explanation of the study in person or over phone by research personnel. REDCap surveys will also be delivered in person or via email/MyChart/text after consent. With each round, after 3-5 patients have participated recruitment will be paused until any necessary modifications to the screening approach are made. Recruitment will resume once the modified approach is implemented. This will occur up to 5 times (n=9-25). **Medical stakeholder** recruitment in the screening approach development and/or the focused interviews will be performed by the research team, with all eligible stakeholders approached on a rolling basis, with purposeful sampling across all roles (provider, nurse, MA). REDCap postcard consent will precede data collection for medical stakeholders. Three to six medical stakeholder surveys will be administered with each round (n=9-30), with purposeful sampling to involve stakeholders of varying roles in the intervention and clinic in general. As compensation, all **stakeholder** survey participants will receive a $10 gift card per event if the local site allows provider incentives.

*Semistructured interviews:* Results of **medical stakeholder** surveys will be reviewed after each round. The research team will attempt to enroll a 1-3 medical stakeholders in each round for participation in a semistructured qualitative interview, first approaching the stakeholder who rated the intervention most critically (n=3-15). Informed consent will precede data collection. As compensation, all **medical stakeholder** interview participants will receive a $40 gift card per interview if the local site allows provider incentives.

*Field notes:* Research personnel will collect field notes to document observations and any additional (e.g., unsolicited or informal) feedback received from patients, families, or healthcare providers or other professionals in the practice environment. No identifiers will be included as part of field notes.

*Focus group*: After completion of the iterative testing and adaptation process, the final SD screening approach will be presented to a focus group of **medical stakeholders** (n=6; a least 1 each of provider, RN, and MA) (Appendix 4). These stakeholders will be recruited from all eligible stakeholders and informed consent will precede focus group participation. Participants will be compensated $40 gift card per focus group if the local site allows provider incentives.

***HYBRID EFFECTIVENESS-IMPLEMENTATION PHASE (Aims 2-3)***

*Pre-Implementation*: After eligibility screening (RA) and review (PI), **patient stakeholders** at each site (CHCO HOPE Survivorship, CHCO Oncology Clinic, CHCO Neuro-Oncology, Seattle Children’s Hospital [SCH] Cancer Survivorship Program) will be approached by research personnel at the end of each visit, or via phone within 4 weeks of the existing appointment, for participation in a survey (n=43). We will use a purposive consecutive sampling to achieve variation across the age categories (age 15-19 and 20-24), patient treatment status (on therapy or survivor), and gender, with final enrollment with a minimum 40% representation for each of these groups. Consent and assent will consist of a paper or REDCap consent/assent with waiver of parental consent and HIPAA waiver for participants <18 years old (when applicable – see section 10.1.1.2), presented in person or via email/MyChart/text after explanation of the study in person or over phone by research personnel. REDCap surveys will also be delivered in person or via email/MyChart/text after consent. Pre-intervention, the survey will assess SD communication and clinical care only (baseline effectiveness), and participation will be compensated with a $10 gift card.

*Post-Implementation:* The screening approach will be implemented across all eligible patients as part of clinical care. The research team will review eligibility of **patient stakeholders** through existing clinic schedules. Eligible patient stakeholders will be approached by research personnel at the end of each visit to present the study (prioritized), or by phone within 4 weeks of eligible visits (SD screening completed or overdue by 4 weeks or more), with a maximum of 3 attempts to reach potential participants. Study team members will be members of the Center for Cancer and Blood Disorder. Consenting/assenting participants completing the survey for data collection prior to leaving the clinic (preferred) or via REDCap emailed/MyChart/text link (n=43). We will again use a purposive consecutive sampling to achieve variation across the age categories (age 15-19 and 20-24), patient treatment status (on therapy or survivor), and gender, with final enrollment with a minimum 40% representation for each of these groups. Consent and assent will consist of a paper or REDCap consent/assent with waiver of parental consent and HIPAA waiver for participants <18 years old (when applicable – see section 10.1.1.2), presented in person or via email/MyChart/text after explanation of the study in person or over phone by research personnel. REDCap surveys will also be delivered in person or via email/MyChart/text after consent. Post-intervention, the survey will both effectiveness (all patients) and implementation (in those who received screening) (Aim 3, below); thus, participation will be compensated with a $20 gift card.

Research personnel will work with the study champions at each site to determine the best method to obtain **medical stakeholder** surveys (e.g., email, paper). Enrollment and consent of medical stakeholders will occur at the end of the post-implementation study phase, with all eligible medical stakeholders approached for participation in surveys (anticipating an 80% response rate, n=16-24). Medical stakeholders will receive $10 compensation for survey completion if the local site allows provider incentives. Medical stakeholder consent will be via REDCap postcard consent, with the opportunity to ask questions to research personnel.

*Semistructured interviews:* Results of medical stakeholder surveys will be reviewed to allow for purposive selection of medical stakeholders for interviews (n=10-15). Research personnel will intentionally approach medical stakeholders with a range of survey responses as well as proportional representation across roles and clinics. Medical stakeholders $40 for interview completion if the local site allows provider incentives. Medical stakeholder consent will be via REDCap postcard consent, with the opportunity to ask questions to research personnel.

# 6 STUDY INTERVENTION

## **6.1 Study INTERVENTION(S) ADMINISTRATION**

### **6.1.1 STUDY INTERVENTION DESCRIPTION**

This study is testing a standardized approach for sexual function screening, which is a part of recommended routine clinical care. The **prototype screening approach,** developed through our prior stakeholder engagement, will be adapted iteratively through stakeholder feedback in Aim 1 of this study. **Following completion of Aim 1, the study protocol will be amended as needed to include any relevant updates to the screening approach.**

The prototype screening approach will consist of:

1. Use of the PROMIS SexFS Brief across all patients ages 15-24 with or surviving cancer with a frequency of every 3 months or at next visit if seen less frequently than every 3 months; there will be a 1-month allowance/buffer from the date due to have screening completed. Flexibility of the intervention will allow each site will determine the appropriate modality to maximize patient privacy, comfort, and completion as well as adherence to HIPAA. Examples of components that may vary by site include modality of deliver (EHR, paper), timing of screening (before, during or after visit), results storage (EHR, paper), exact frequency of delivery, provider management (responsible providers, clinical resources).
2. Implementation approach: All sites will be provided with recommended strategies for implementation, which will be refined in Aim 1. Example strategies include use of provider education, clinical champions, EHR reminders, provider resources, and patient resources.

## **6.2 study intervention compliance**

Completion and use of the screening approach will be measured as key implementation outcomes via Aim 3. Specifically, we will measure:

1. Reach via record abstraction evaluating the proportion of eligible patients who completed SD screening and provider response on reasons for non-completion within 1 month of due.
2. Adoption via survey score of a 5-point scale on a survey at the conclusion of the study, asking (a) how often medical stakeholders reviewed SD screening results with relevant patients and (b) how often they discussed results with patients; a secondary measure of adoption will be results views by the provider (i.e. electronic health record (EHR) clicks)

# 7 STUDY INTERVENTION DISCONTINUATION AND PARTICIPANT DISCONTINUATION/WITHDRAWAL

## **7.1 discontinuation of study intervention (Study stopping rules)**

The Sponsor-Investigator has the right to terminate this study at any time. Reasons for terminating the study may include, but are not limited to, the following:

- The incidence or severity of adverse events in this or other studies indicates a potential health hazard to patients.
- Patient enrollment is unsatisfactory.

## **7.2 PARTICIPANT DISCONTINUATION/WITHDRAWAL FROM STUDY**

Participants are free to withdraw from participation in the study at any time upon request.

In addition, the investigator has the right to withdraw a patient from the study at any time. Reasons for withdrawal from the study may include, but are not limited to, the following:

- Patient withdrawal of consent/assent at any time
- Any medical condition that the Sponsor-Investigator determines may jeopardize the patient’s safety if he or she continues in the study.
- Sponsor-Investigator determines it is in the best interest of the patient.
- Patient non-compliance

Patients must discontinue study intervention if they experience any of the following:

- Discomfort
- Inability to complete surveys for any reason

Every effort should be made to obtain information on patients who withdraw from the study. The primary reason for withdrawal from the study should be documented on the appropriate eCRF.

## **7.3 lost to follow-up**

**Patient stakeholders** will be asked to complete surveys during their existing clinic visits immediately after consent/assent and enrollment. However, consent/assent, enrollment and surveys may be completed within 4 weeks of eligible visits (when sexual function screening are due).

# 8 STUDY PROCEDURES

## **8.1 study procedures/evaluations**

***ADAPTATION PHASE (Aim 1)***

**Study Design:** The prototype screening approach developed in prior studies will be implemented in the CHCO Neuro-Oncology clinic and undergo a multi-stage iterative test and revision process, guided by end-user feedback. We will test the screening approach through surveys on acceptability, appropriateness and feasibility with all participating patient and provider stakeholders. Surveys will consist of brief demographic data collection and adapted versions of three brief 4-item measures: Acceptability of Implementation Measure (AIM), Implementation Appropriateness Measure (IAM), and Feasibility of Intervention Measure (FIM) (details below and in Appendix 2), in conjunction with specific questions to elicit CFIR barriers/facilitators. We will also perform key informant interviews with a subset of medical stakeholders who score high and low on acceptability, appropriateness and feasibility surveys (interview guide available in Appendix 3). Field notes will also be collected by the research team.

Results will inform modifications to the screening approach by the research team, informed by stakeholder recommendations and use of the CFIR-ERIC (Expert Recommendations for Implementing Change) matching tool, which links CFIR barriers with implementation strategies to mitigate these barriers. Based on recommendations in the literature, we expect that this process will require 3-5 rounds of iterative modifications addressing stakeholder feedback. Each round will consist of 3-5 patient interviews, 3-6 medical stakeholder surveys, and 1-3 medical stakeholder interviews (total unique patients=9-25, medical stakeholder surveys=9-30 and interviews=3-15).^50^ Three stopping criteria will be used:

1. Saturation in key informant interviews regarding identified barriers/facilitators (i.e., no new unaddressed barriers/facilitators identified)
2. At least 80% of each group of stakeholders (patient, medical) surveyed will have scores of 4 or higher on the AIM, FIM, and IAM (indicating an average response of “agreeing” or “strongly agreeing” to items assessing the acceptability, feasibility, and appropriateness of the screening approach)
3. Stakeholders will identify no major problems with the screening approach (note that in the user-centered design field, “major problems” are defined as those which would severely limit the ability of end-users to implement the screen approach).

Once these stopping rules are met, the PI, mentors, and collaborators will produce a draft screening approach protocol and implementation package (e.g., use of a “clinical champion”, provider training, use of reminders in the electronic health record (EHR), flexibility for electronic or paper administration). If the stopping rules have not been met after 5 rounds, the research team will decide upon the screening approach protocol and implementation package based on feedback elicited through the 5th round.

Finally, a stakeholder focus group (n=6), recruited from prior medical stakeholders, will be convened to review the draft screening approach protocol (Appendix 4). Following this final feedback elicitation, the research team will produce the final screening approach protocol, implementation materials, and a Fidelity Checklist.

**Measures**: As outlined above, data collection will occur in multi-stage iterative testing via brief surveys and purposive focused interviews with stakeholders, focusing on RE-AIM implementation, acceptability, and feasibility, and potential CFIR-guided barriers and facilitators to these outcomes. Surveys will consist of three brief adapted 4-item measures: Acceptability of Implementation Measure (AIM), Implementation Appropriateness Measure (IAM), and Feasibility of Intervention Measure (FIM) (Appendix 2), in conjunction with specific questions to elicit CFIR barriers/facilitators. AIM/IAM/FIM are freely available and have demonstrated content and structural validity and test-retest reliability.^2^ CFIR-guided questions will elicit contextual factors in CFIR domains: 1) intervention characteristics (e.g., work flow compatibility), 2) outer setting (e.g., external policy), 3) inner setting (e.g., organizational culture), 4) individual characteristics (e.g., knowledge and beliefs about the intervention, provider role), and 5) process (e.g., key personnel). Qualitative interviews will inquire reasoning for respondents’ ratings as well as further explore CFIR factors (Appendix 3). Brief demographic data will also be collected for each participant.

**Data Collection**: Survey collection will occur in person at the end of patient encounters by the research team. Explanatory qualitative interviews will occur in person or by phone with medical stakeholders, by a trained qualitative research assistant (RA); these will be digitally recorded and professionally transcribed. Surveys and interview guides will be developed following the CFIR Interview Guide and will include items such as ease of use, compatibility with current clinic practices, and helpfulness/benefit. Iterations of the screening approach will be documented through detailed notes.

***HYBRID EFFECTIVENESS-IMPLEMENTATION PHASE (Aims 2-3)***

**Study Design:** We will use a pre-post hybrid effectiveness-implementation study design at 4 clinical sites.

Baseline, pre-implementation data collection will occur before the iterative intervention development in Aim 1 to avoid contamination. Following baseline data collection, the SD screening approach developed in Aim 1 will be implemented in three clinics and effectiveness data will be prospectively collected. All four sites will receive the intervention clinic-wide (for all patients meeting study criteria) and will contribute data in both the pre- and post-intervention phases. Thus, participants are part of a predefined group that will be “assigned” to receive the intervention by receiving care at the clinics where the intervention is being implemented. Prior to implementation of the screening approach, all relevant providers will be trained on the screening approach via a virtual training session, and any necessary practice-specific modifications will be made. Implementation strategies will be refined in Aim 1, but may include establishing clinical champions, EHR reminders, provider resources, and patient handouts.

In conjunction with Aim 2, we will use an explanatory sequential mixed methods design to measure implementation outcomes at each site during the post-intervention period. Quantitative data will be collected via surveys; patient stakeholder (n=43) outcomes will be assessed in real-time, within 1 month of screening completed/overdue during the implementation phase, whereas medical stakeholders (n=16-24) will be assessed post-implementation to avoid biasing providers while implementation of the screening approach continues. Quantitative analyses of implementation outcome data will be followed by a qualitative assessment to explain survey results, including exploration of multilevel barriers and facilitators to implementation via purposive interviewing with high- and low-rating stakeholders (n=10-15). At sites where only certain medical stakeholders are responsible for SD screening/follow-up, additional brief interviews assessing acceptability will be completed (n=5-10).

**Data Collection/Outcomes*:***

*Effectiveness*: Data will be collected prospectively on patients during the 3-month pre-intervention phase (n=43) and patients who receive SD screening within 1 month of it being due during the 3-month post-intervention phase (n=43) (total n=86). Additional data will be collected EHR, including documentation of and referrals for SD. Participants will complete a survey (Appendix 5) at the end of scheduled visits or via phone within 4 weeks of screening completion. The **primary outcome** is a patient-reported indicator that his/her provider has communicated with them about SD. We anticipate that most providers will only address SD with patients who report a concern on the SexFS Brief; this assumption is reflected in sample size calculations. However, because our preliminary work demonstrated that patients wanted SD conversations to accompany the SexFS Brief, our analysis will include all patients, regardless of SD screening results. **Secondary outcomes**, detailed in section 3, include patient satisfaction with SD communication^51^ and patient-report that an SD need was met. We will also assess the feasibility of collecting EHR data in all eligible patients via retrospective chart review (exploratory outcomes):

- Clinic/site
- Incidence of documented/detected SD
- Referral patterns to SD-related specialty care (fertility team, urology, gynecology/oncology, sexual health clinic, endocrinology, etc.) via EHR order or note documentation **(exploratory outcomes)**.

These secondary and exploratory outcomes will inform a future multicenter trial defining the clinical impact of our screening intervention. Brief demographic data will also be collected for each participant.

*RE-AIM Outcomes:* Data will be collected using a combination of quantitative patient (Appendix 5) and medical provider surveys (Appendix 6) purposive qualitative interviews with medical stakeholders, and research personnel observation (Appendix 7 and 8).^2,52^ For sites that choose to have a limited number of providers responsible for survey delivery and follow-up/management, only responsible providers will receive complete surveys. Surveys will be administered in person by the RA. Interviews will be completed in person or via telephone by the study’s qualitative RA using CFIR interview guides, similar to Aim 1.^48^

Details on assessment:

*Reach:* Reach will be assessed primarily through record abstraction evaluating the proportion of eligible patients who completed SD screening within 1 month of it being due. Secondarily, for patients who do not receive screening within 1 month of it being due, reach will be further explored via a single multiple choice question (Appendix 9) to the medical team inquiring into the reasoning for missed screening. *Representativeness* will be assessed by comparing sociodemographic characteristics between eligible patients who did versus did not receive screening (reported in aggregate/anonymously, corrected during the study eligibility screening process).

*Adoption:* **Medical stakeholder** adoption will be assessed via survey score of a 5-point scale on a survey at the conclusion of the study, asking (a) how often they reviewed SD screening results with relevant patients and (b) how often they discussed results with patients. If the EHR is used to deliver/report the survey (determined in Aim 1), we will explore objective measures, such as whether the results were viewed by the provider (i.e., clicks).

*Implementation:* Implementation will be assessed using four of Proctor’s implementation outcomes: *acceptability, appropriateness, feasibility and fidelity.^53^* These implementation outcomes will be measured from the perspectives of both patients and medical stakeholders, as well as via observation by the RA. **Medical stakeholder** outcomes will be measured at the conclusion of the study using the adapted AIM, IAM and FIM surveys in providers who are involved in SD screening/management; these surveys will also include basic demographic information (Appendix 6).^2^ At sites where there is a set group of responsible providers, acceptability will also be assessed among non-responsible providers in brief interviews (Appendix 8) **Patient stakeholders** who received the screening will complete patient**-reported** outcomes measures (5-point scale questions adapted from the AIM, IAM, and FIM (Appendix 5), as well as by the Fidelity Checklist developed in Aim 1).^52^ The Fidelity Checklist will also be completed through direct study personnel observation of the screening approach in 10% of patients across sites.^52^ Finally, study personnel will document adaptations to the screening approach as they occur throughout the study.

*Contextual Factors:* Guided by CFIR and using an explanatory mixed method design, we will complete 10-15 purposive interviews with **medical stakeholders** who rated the appropriateness, acceptability, or feasibility high or low to better understand contextual factors that serve as barriers or facilitators to use (Appendix 7). For example, to better understand feasibility, we will ask, “How did review of the screening tool fit into your workflow?” and follow up with direct questions about barriers, engagement, perceived successes/failures, and recommendations for improvement. Results will (a) help explain any observed variation in implementation outcomes across the three sites, and (b) inform selection or development of implementation strategies, informed by the CFIR-ERIC matching tool, to promote implementation across a variety of settings in our subsequent large-scale multisite trial.^54^

**Potential variables will be collected on consenting/assenting patients:**

- Survey-collected data (patient report):
  - Gender identity
  - Age
  - Race
  - Gender
  - Ethnicity
  - Treatment/disease status
- Medical record extraction
  - Clinic/site
  - Presence/absence and type of endocrinopathy
  - Exposure and dose of alkylator chemotherapy
  - Prior history of spinal, pelvic or testicular radiation
  - Prior history of spinal or pelvic surgery
  - Documentation of sexual function concerns (before and after screening)
  - Treatment for sexual function concerns (before and after screening)

## **8.2 ADVERSE EVENTS AND SERIOUS ADVERSE EVENTS**

### **8.2.1 Definition of Adverse Events (AE)**

**Adverse event** means any untoward medical occurrence associated with the use of an intervention in humans, whether or not considered intervention-related.

### **8.2.2 Definition of Serious Adverse Events (SAE)**

**Serious adverse event** or **serious suspected adverse reaction**. An AE or suspected adverse reaction is considered “serious” if, in the view of either the investigator or sponsor, it results in any of the following outcomes: death, a life-threatening adverse event, inpatient hospitalization or prolongation of existing hospitalization, a persistent or significant incapacity or substantial disruption of the ability to conduct normal life functions, or a congenital anomaly/ birth defect. Important medical events that may not result in death, be life-threatening, or require hospitalization may be considered serious when, based upon appropriate medical judgment, they may jeopardize the patient or subject and may require medical or surgical intervention to prevent one of the outcomes listed in this definition.

### **8.2.3 CLASSIFICATION OF AN ADVERSE EVENT**

### **8.2.3.1 severity of event**

For AEs, the following guidelines will be used to describe severity.

- **Mild** – Events require minimal or no treatment and do not interfere with the participant’s daily activities.
- **Moderate** – Events result in a low level of inconvenience or concern with the therapeutic measures. Moderate events may cause some interference with functioning.
- **Severe** – Events interrupt a participant’s usual daily activity and may require systemic drug therapy or other treatment. Severe events are usually potentially life-threatening or incapacitating.

| Grade Severity | |
| --- | --- |
| 1. Mild; asymptomatic or mild symptoms; clinical or diagnostic observations only; or intervention not indicated 2. Moderate; minimal, local, or non-invasive intervention indicated; or limiting age-appropriate instrumental activities of daily living a 3. Severe or medically significant, but not immediately life-threatening; hospitalization or prolongation of hospitalization indicated; disabling; or limiting self-care activities of daily living b,c 4. Life-threatening consequences or urgent intervention indicated 5. Death related to adverse event |  |

NCI CTCAE = National Cancer Institute Common Terminology Criteria for Adverse Events. Note: Based on the most recent version of NCI CTCAE (v5.0), which can be found at: <http://ctep.cancer.gov/protocolDevelopment/electronic_applications/ctc.htm>

a Instrumental activities of daily living refer to preparing meals, shopping for groceries or clothes, using the telephone, managing money, etc.

b Examples of self-care activities of daily living include bathing, dressing and undressing, feeding oneself, using the toilet, and taking medications, as performed by patients who are not bedridden.

c If an event is assessed as a "significant medical event," it must be reported as a serious adverse event.

### **8.2.3.2 Relationship to Study INTERVENTION**

Only adverse events directly related to study intervention will be recorded.

### **8.2.3.3 Expectedness**

Expectedness will only be documented for SAEs. The PI will be responsible for determining whether an SAE is expected or unexpected. An SAE will be considered unexpected if the nature, severity, or frequency of the event is not consistent with the risk information previously described for the study intervention.

### **8.2.4 time period and frequency for event assessment and follow-up**

The occurrence of an AE or SAE may come to the attention of study personnel during study visits and interviews of a study participant presenting for medical care, or upon review by a study monitor. All AEs including local and systemic reactions not meeting the criteria for SAEs will be captured on the appropriate CRF. Information to be collected includes event description, time of onset, clinician’s assessment of severity, (if applicable) relationship to study intervention (assessed only by those with the training and authority to make a diagnosis), and time of resolution/ stabilization of the event. All AEs occurring while on study must be documented appropriately. All AEs will be followed to adequate resolution.

Any medical condition that is present at the time that the participant is screened will be considered as baseline and not reported as an AE. However, if the study participant’s condition deteriorates at any time during the study, it will be recorded as an AE. UAPs will be recorded in the data collection system throughout the study.

Changes in the severity of an AE will be documented to allow an assessment of the duration of the event at each level of severity to be performed. AEs characterized as intermittent require documentation of onset and duration of each episode.

The PI will record all reportable events with start dates occurring any time after informed consent/assent is obtained until 30 days after the last day of study treatment or until initiation of new treatment, whichever comes first. SAEs will be followed until resolution or stabilization. At each study visit, the investigator will inquire about the occurrence of AE/ SAEs since the last visit.

### **8.2.5 Adverse Event Reporting**

The investigator must record non-serious adverse events and report to DSMC and IRB according to timetable for reporting specified in section 10.1.6.

### **8.2.6 serious adverse event reporting**

The investigator must record all serious adverse events and report to DSMC and IRB according to timetable for reporting specified in section 10.1.5. Per institutional policy, all serious adverse events occurring at UCCC/UCHealth will be entered into OnCore according to timetable for reporting specified in section 10.1.5.

All SAEs will be reported using the FDA 3500A Mandatory MedWatch report form unless funding sponsor or drug manufacturer requires use of proprietary form. SAE form can be found at: <http://www.fda.gov/downloads/AboutFDA/ReportsManualsForms/Forms/UCM048334.pdf>

To submit an SAE, email as follows within 24 hours of becoming aware of the event:

To: jenna.Demedis@cuanschutz.edu

[cpdm.iit@cuanschutz.edu](mailto:cpdm.iit@cuanschutz.edu)

[DSMC@cuanschutz.edu](mailto:DSMC@cuanschutz.edu)

Subject: 22-0709 SAE Report Form

Attach: SAE form completed and signed by the Investigator

**Follow-up of unresolved serious adverse events**

Any SAEs that are unresolved at the time of the initial report submission should be followed up by the investigator for as long as medically indicated, and an updated SAE report submitted at the time new information regarding the event becomes available.

## **8.3 UNANTICIPATED PROBLEMS**

### **8.3.1 Definition of Unanticipated Problems (UAP)**

The Office of Human Research Protection (OHRP) considers unanticipated problems involving risks to participants or others to include, in general, any incident, experience, or outcome that meets **all** of the following criteria:

- Unexpected in terms of nature, severity, or frequency given (a) the research procedures that are described in the protocol-related documents, such as the IRB-approved research protocol and informed consent/assent document; and (b) the characteristics of the participant population being studied;
- Related or possibly related to participation in the research (“possibly related” means there is a reasonable possibility that the incident, experience, or outcome may have been caused by the procedures involved in the research); and
- Suggests that the research places participants or others at a greater risk of harm (including physical, psychological, economic, or social harm) than was previously known or recognized.

This study will use the OHRP definition of UAP.

### **8.3.2 REPORTING of Unanticipated Problems**

Incidents or events that meet the OHRP criteria for UAPs require the creation and completion of a UAP report. It is the Site PI’s responsibility to report UAPs to their IRB. The Lead PI is responsible for reporting the UAP to the IRB and the UCCC DSMC. The UAP report will include the following information:

- Protocol-identifying information: protocol title and number, PI’s name, and the IRB project number;
- A detailed description of the event, incident, experience, or outcome;
- An explanation of the basis for determining that the event, incident, experience, or outcome represents a UAP;
- A description of any changes to the protocol or other corrective actions that have been taken or are proposed in response to the UAP.

# 9 statistical considerations

## **9.1 Aim 1 Statistical plan**

**Aim 1. Develop and refine a routine, standardized SD screening approach for implementation in a pediatric oncology clinic using stakeholder input and iterative design.**

Endpoints

Analytic Plan: Facilitators and barriers identified in quantitative surveys and qualitative interviews will be integrated by mapping to the CFIR framework in a team approach (PI, methods mentors and qualitative research assistant). Initial deductive rapid coding will be performed by the research team via listening to recordings while completing the summary template and following a coding guide. This approach uses characteristics and content of language to provide understanding of qualitative data through systematic classification, coding, and theme identification. Coding will involve interpreting and assigning data into the Consolidated Framework for Implementation Research (CFIR) coding schema; for example, “lack of time” will be coded as an “inner setting barrier.” To enhance rigor, the two members of the research team will each independently review an initial set of recordings and meet to compare results and resolve discrepancies. This process will continue until discrepancies are minimal, at which point the research team will independently code the remainder of recordings. Results will inform modifications to the screening approach by the research team, informed by stakeholder recommendations and use of the CFIR-ERIC (Expert Recommendations for Implementing Change) matching tool, which links CFIR barriers with implementation strategies to mitigate these barriers. Based on recommendations in the literature, we expect that this process will require 3-5 rounds of iterative modifications addressing stakeholder feedback. Three stopping criteria will be used: (1) saturation in key informant interviews (no new unaddressed barriers/facilitators); (2) survey results indicate at least 80% acceptability, feasibility and appropriateness of the screening approach; and (3) stakeholders will identify no major problems with the screening approach (defined as those which would severely limit the ability of end-users to implement the screen approach). Once stopping rules are met, the research team will produce a draft screening approach and implementation package (e.g., use of a “clinical champion”, provider training, use of reminders in the electronic health record (EHR)). If the stopping rules have not been met after 5 rounds, the research team will decide upon the screening approach protocol and implementation package based on all elicited feedback. Changes will be documented in a “development document,” including dates, explanations, and discussion notes. Iterative changes will continue via the above process, with attention paid to preferences by sex/gender. The digital recordings will be later transcribed, and the same qualitative team will analyze transcripts using formal content analysis, which will further guide implementation planning. Finally, a stakeholder focus group (Appendix 4), recruited from prior medical stakeholders, will be convened to review the draft screening approach protocol. Following this final feedback elicitation, the research team will produce the final screening approach protocol, implementation materials, and a Fidelity Checklist.

Sample Size: We anticipate 3-5 rounds of pretesting and modification over 6 months, with each round consisting of 3-5 patient interviews, 3 medical stakeholder surveys, and 1 medical stakeholder interview (total unique patients=9-25, medical stakeholder surveys=9-15 and interviews=3-5). There are 9 eligible providers, 3 eligible nurses and 1 eligible MA; over the course of Aim 1, we will attempt to survey all providers/nurses/MAs at least once and perform interviews across each provider type. Though there is no consensus in the user-centered design literature on required sample size, we anticipate that our planned total sample size (n=27-51) for the iterative feedback and modification process, combined with our stopping criteria, are sufficient for our purpose and scope. For the final stakeholder focus group, we will 6 neuro-oncology medical stakeholders with at least 1 each of provider, RN, MA.

## **9.2 AIM 2 Statistical plan**

**Aim 2. Evaluate the preliminary effectiveness of the standardized screening approach for SD on occurrence of patient-provider SD communication.**

Analytic Plan: Descriptive statistics (means, medians, standard deviations, frequencies) will be computed for patient characteristics, overall and stratified by study period. Differences in patient predictors between study periods will be assessed using chi-squared and t-tests (or appropriate bivariable tests based on variable type and distribution).

The primary analysis will compare the effect of the intervention on the proportion of patients that self-report SD screening. We will use a multivariable logistic regression model for the binary outcome of self-reported SD screening, controlling for patient-level predictors identified a priori to be potential confounders (e.g., age, gender, presence of SD concerns, endocrinopathies, clinic site, on therapy or survivor). Although there is correlation due to the hierarchical structure of the data (patients within sites), due to the limited number of sites (<5), we will include a fixed effect for site rather than a random effect. The specific model will be of the form:

η_i_ = β_0_ + β_1_T_i_ + β_2_’Z_i_, (1)

where η_i_= log (ϕ_i_/ (1 - ϕ_i_)), is the log odds of success for patient i (i.e., patient i self-reported SD screening), T_i_ is an indicator of whether the measurement was taken in the pre-intervention (T_i_=0) or post-intervention (T_i_=1) period, Z_i_ is a vector of patient-level predictors including the site at which they were seen. To assess the intervention effect, we test the null hypothesis H_0_: β_1_=0 vs. H_1_: β_1_ ≠ 0. We will perform a subgroup analysis by key variables (site, therapy/survivor status) using an interaction between the subgroup variable and the intervention period. The secondary outcome of whether a patient reported that their SD need was met will be analyzed similarly, overall and in the subgroup of those that indicated that they had an SD need, which will be assessed using an interaction with the SD need indicator and the pre-/post- indicator.

We will also use similar multivariable regression models adjusted for the same covariates to assess the effect of the intervention on secondary and exploratory outcomes. The secondary outcome of patient satisfaction measured on a 0-10 scale will be modelled as a continuous outcome using a multivariable linear regression model. The exploratory outcome of feasibility of collecting EHR data will be modelled as a binary outcome (yes/no documented/detected SD) using a logistic regression model. The exploratory outcome of number of referral patterns will be modelled as a count outcome using a multivariable Poisson regression model. To examine whether the intervention effect differs by key variables (site, therapy/survivor status) we will assess for an interaction effect between study period (pre/post) and the variable.

Goodness-of-fit tests and model-fitting diagnostics will be performed for proposed analyses to assess for influential points, outliers, and to evaluate alternative model specifications. All hypothesis tests will be two-sided with alpha=0.05, and p-values and confidence intervals will be reported. Statistical analyses will be conducted using R or SAS version 9.4 (SAS Institute Inc., Cary, N.C.).

Power: Sample size calculations are based on the number of participants needed pre- and post-implementation of the screening approach to detect a 25% difference in proportion of patients reporting SD conversations with their providers (5% pre-intervention vs. 30% post-intervention), aggregated across all sites. Assuming a multivariable logistic regression in which 20% of the variability in the independent variable is explained by the other predictors, to detect a clinically relevant difference of 25% (5% pre-intervention vs. 30% post-intervention) in SD conversations with a two-sided test, 80% power, and alpha =.05, 86 participants will be required (43 pre- and 43 post-implementation of screening approach).

This power calculation is based on the following assumptions: 1) Pre-implementation, we anticipate 5% of participants will report having had an SD conversation with their providers (conservative, based on prior literature). 2) Post-implementation, we estimate that 30% of patients will report an SD conversation with their provider (conservative, based on (a) estimated SD prevalence rates of 20-50% and (b) the assumption that some patients/providers will initiate conversations regardless of SexFS Brief results). Assuming a conservative 50% response rate and approximately 38 eligible patients per month (derived from 2019 clinical volume), we anticipate that recruiting 43 patients for each study period will require approximately 3 months (0.5*38*3=57).

## **9.3 aim 3 Statistical plan**

**Aim 3. Assess preliminary implementation outcomes and contextual factors associated with implementation of the standardized screening approach for SD.**

Analytic Plan: Because this is a pilot type 1 hybrid trial, analyses of implementation outcomes and factors in Aim 3 are descriptive, aiming to inform a future multicenter randomized hybrid trial. Using an explanatory mixed method design, we will first use descriptive statistics to analyze **quantitative survey data**, which will inform qualitative data collection. All quantitative analyses will be performed by the study statistician, with data summarized for the entire cohort, as well as by site, on therapy or survivor, patient gender, patient age and provider type. **Qualitative data analysis** be performed by the qualitative/D&I research and reviewed by the entire research team. Qualitative analysis will follow rapid content analysis methods and will integrate the CFIR framework, as described in Aim 1. Codes and quantitative results will be summarized in a joint matrix to support development of implementation strategies to strengthen future testing and delivery of the screening approach.

Sample Size: As a type 1 hybrid trial, this study is powered on Aim 2, but will explore preliminary implementation outcomes. Aim 3 **patient stakeholder** surveys will be administered in conjunction with Aim 2 post-implementation surveys to patients who received screening (maximum n=43). As this aim measures preliminary/exploratory implementation and is limited to those who received screening, we will not have a minimum sample size. Reasons for missed screening will be collected separately from providers. Aim 3 **medical stakeholder** surveys will be administered to consenting providers (anticipating an 80% response rate, approx. n=16-24), and Aim 3 semi-structured interviews with be completed with 10-15 providers directly involved in the screening intervention. At sites where only certain medical stakeholders are responsible for SD screening/follow-up, additional brief interviews assessing acceptability will be completed (n=5-10). Because the qualitative interviews will be focused on experiences with the screening approach and barriers/facilitators, we anticipate reaching thematic saturation with these sample sizes.^50^ User-centered design and qualitative research findings demonstrate that on average, 5 users/interviews will identify around 73-85% of all problems, and that groups of 10 find 95% of all problems (reaching near-saturation) when evaluating a design.^50,55^

# 10 SUPPORTING DOCUMENTATION AND OPERATIONAL CONSIDERATIONS

## **10.1 regulatory, ethical, and study oversight considerations**

### **10.1.1 informed consent/Assent process**

### **10.1.1.1 Consent/assent and Other Informational Documents Provided to Participants**

Consent/assent forms describing in detail the screening intervention, study procedures, and risks are given to the participant and written documentation of informed consent/assent is required prior to starting intervention/ administering study product.

Seattle Children’s Hospital and Children’s Hospital Colorado have different consents. Seattle Children’s Hospital does not allow compensation for provider participation whereas CHCO does. The Seattle ICF clarifies that there will be no cost to neither the participant nor the participant’s insurance for the research procedures and added their own required HIPAA language. Seattle ICFs clarifies that information is kept confidential.

### **10.1.1.2 Consent/assent Procedures and Documentation**

Informed consent/assent process will be initiated prior to the individual’s agreeing to participate in the study and continues throughout the individual’s study participation. Extensive discussion of risks and possible benefits of participation will be provided to the participants and their families.

Consent and assent forms will be IRB-approved and the participant will be asked to read and review the document. The investigator will explain the research study to the participant and answer any questions that may arise. All participants will receive a verbal explanation in terms suited to their comprehension of the purposes, procedures, and potential risks of the study and of their rights as research participants. Participants will have the opportunity to carefully review the written consent/assent form and ask questions prior to signing. The participants will have the opportunity to discuss the study with their surrogates or think about it prior to agreeing to participate. The participant will sign the informed consent/assent document prior to any procedures being done specifically for the study.

The participants may withdraw consent/assent at any time throughout the course of the trial. A copy of the informed consent/assent document will be given to the participants for their records. The rights and welfare of the participants will be protected by emphasizing to them that the quality of their medical care will not be adversely affected if they decline to participate in this study.

All Aims:

Informed consent/assent process will be initiated prior to the individual's agreeing to participate in the study and continues throughout the individual's study participation. Discussion of risks and possible benefits of participation will be provided to the participants in a private setting. The strictly voluntary nature of the study will be clearly stated in recruitment materials or invitations and in the verbal consent for stakeholder interviews. All participants will be provided with information about the study and researcher's contact information.

Consent and assent forms will be IRB-approved and the participant will be asked to read and review the document. The investigator will explain the research study to the participant and answer any questions that may arise. All participants will receive a verbal explanation in terms suited to their comprehension of the purposes, procedures, and potential risks of the study and of their rights as research participants. Participants will have the opportunity to carefully review the written consent/assent form and ask questions prior to participating in study procedures. The participants will have the opportunity to discuss the study with whomever they wish, and to think about it prior to agreeing to participate.

The participants may withdraw consent/assent at any time throughout the course of the trial. The rights and welfare of the participants will be protected by emphasizing to them that the quality of their medical care will not be adversely affected if they decline to participate in this study.

Medical stakeholder surveys, focus groups, and interviews (Aims 1 and 3): Study presentation by the study personnel through existing professional relationships between potential participants and site PIs. Consent for study and HIPAA will be obtained via REDCap. Importantly, participation confers no greater than minimal risk to participants.

Clinical Trial (Aims 2-3): The screening approach will be implemented clinic-wide, across all eligible patients as a new clinical care practice. This will be done by the clinic staff after appropriate training on use the screening approach. This is acceptable because it is not outside of usual care, which includes discussions of sexual health between clinical providers and patients, and because risks are minimal.

Patient stakeholder surveys and EHR data collection (Aims 1 and 2): Because the study and site PIs have existing clinical relationships with all eligible patients, waiver of HIPAA authorization will not be necessary prior to eligibility screening. Eligible patients will be presented the study by the study team at the conclusion of existing clinic visits or via phone within 4 weeks of eligible visits. Study team members contacting patients for study presentation will be members of the Center for Cancer and Blood Disorder.

As has been done in our prior research, to maximize privacy/confidentiality given the sensitive topic, and to encourage honest participation, patient participation in surveys (Aims 1-3) and EHR data collection (Aim 2-3) will occur under a waiver of parental consent. *Justification for waiver of parental consent:* For both the protection of the participant and the quality of the study parental/guardian consent will not be required. Given the low risk associated with participation in a survey study, we request parental consent to be waived for minor participants. This study poses no more than minimal risk (no greater than that encountered within routine history and physical examination). Inability to administer this survey without parental consent will severely bias the findings: 1) The sample may be biased as only those patients most comfortable with sexual health discussions (as those they might have with their parents) would be willing to participate if parental consent were required, and 2) Data collection may be biased by requiring parental consent, as adolescents may be concerned about confidentiality/safety. Subjects must be reassured that information they share, both in completing the screening approach in clinic and in completing research surveys, will be kept confidential and that participation itself is confidential.

### **10.1.2 STUDY DISCONTINUATION AND CLOSURE**

N/A

### **10.1.3 confidentiality and privacy**

Participant confidentiality is strictly held in trust by the participating investigators, their staff, and the sponsor-investigator(s) and their agents. This confidentiality is extended to cover testing of biological samples and genetic tests in addition to the clinical information relating to participants. Therefore, the study protocol, documentation, data, and all other information generated will be held in strict confidence. No information concerning the study or the data will be released to any unauthorized third party without prior written approval of the sponsor.

The study monitor, other authorized representatives of the sponsor-investigator, or representatives of the IRB may inspect all documents and records required to be maintained by the investigator, including but not limited to, medical records (office, clinic, or hospital) for the participants in this study. The clinical study site will permit access to such records.

The study participant’s contact information will be securely stored at each clinical site for internal use during the study. At the end of the study, all records will continue to be kept in a secure location for as long a period as dictated by local IRB and Institutional regulations.

Study participant research data, which is for purposes of statistical analysis and scientific reporting, will be transmitted to and stored at University of Colorado ACCORDS center. This will not include the participant’s contact or identifying information. Rather, individual participants and their research data will be identified by a unique study identification number. The study data entry and study management systems used by clinical sites and by the University of Colorado ACCORDS center research staff will be secured and password protected. At the end of the study, all study databases will be de-identified and archived at Children’s Hospital Colorado and the University of Colorado ACCORDS center.

### **10.1.4 future Use of Stored Specimens or Data**

- **Intended Use:** Samples and data collected under this protocol may be used to study sexual health screening and patient-reported outcome use. No genetic testing will be performed.
- **Storage:** Data will be stored using codes assigned by the investigators. Data will be kept in password-protected computers. Only investigators will have access to the samples and data.
- **Tracking:** Data will be tracked using REDCap.

### **10.1.5 safety oversight**

**Non-Interventional IIT (No Additional Risk) At Multiple institutions**

The principal investigator will be responsible for the conduct of this study at all participating institutions, overseeing participant safety, executing the data and safety monitoring (DSM) plan, and complying with all reporting requirements to local and federal authorities. This oversight will be accomplished through additional oversight from the Data and Safety Monitoring Committee (DSMC) at the University of Colorado Cancer Center (CU Cancer Center). The DSMC is responsible for ensuring data quality and study participant safety for all trials at the CU Cancer Center. A summary of the DSMC’s relevant activities is as follows:

- Conduct of internal audits
- May submit recommendations for corrective actions to the CU Cancer Center’s Executive Committee

Study audits conducted by the DSMC will consist of a review of the regulatory documents, consent and assent forms, and source data verification. Documentation of the audit conducted by the DSMC will then need to be submitted to the IRB of record at the time of the IRB’s continuing review of this trial (if applicable).

### **10.1.6 clinical monitoring**

Clinical site monitoring will be conducted to ensure that the rights and well-being of human participants are protected, that the reported trial data are accurate, complete, and verifiable, and that the conduct of the trial is in compliance with the currently approved protocol/ amendment(s), with GCP, and with applicable regulatory requirement(s).

Monitoring for this study will be performed by CU Cancer Center Clinical Monitor in accordance with the clinical monitoring plan (CMP), incorporated herein by reference. The CMP describes in detail who will conduct the monitoring, at what frequency monitoring will be done, at what level of detail monitoring will be performed, and the distribution of the monitoring reports.

### **10.1.7 quality assurance and quality control**

Quality Control (QC) procedures will be implemented beginning with the data entry system and data QC checks that will be run on the database will be generated. Any missing data or data anomalies will be communicated to the site(s) for clarification/ resolution.

Following written SOPs, the study monitor will verify that the clinical trial is conducted, and data are generated, documented (recorded), and reported in compliance with the protocol, GCP, and the applicable regulatory requirements.

The investigational site will provide direct access to all trial-related sites, source data/ documents, and reports for the purpose of monitoring and auditing by the DSMC audit team, and inspection by local and regulatory authorities.

### **10.1.8 data handling and record keeping**

### **10.1.8.1 data collection and management responsibilities**

Data collection is the responsibility of the clinical trial staff at the site under the supervision of the site PI. The PI is responsible for ensuring the accuracy, completeness, legibility, and timeliness of the data reported.

All source documents should be completed in a neat, legible manner to ensure accurate interpretation of data. When making changes or corrections, cross out the original entry with a single line, and initial and date the change. DO NOT ERASE, OVERWRITE, OR USE CORRECTION FLUID OR TAPE ON THE ORIGINAL.

Copies of the electronic CRF (eCRF) will be provided for use as source documents and maintained for recording data for each participant enrolled in the study. Data reported in the eCRF derived from source documents should be consistent with the source documents or the discrepancies should be explained and captured in a progress note and maintained in the participant’s official electronic study record.

Clinical data (including AEs, concomitant medications, and expected adverse reactions data) and clinical laboratory data will be entered into REDCap and OnCore. The data system includes password protection and internal quality checks, such as automatic range checks, to identify data that appear inconsistent, incomplete, or inaccurate. Clinical data will be entered directly from the source documents.

### **10.1.8.2 study records retention**

Study documents should be retained for a minimum of 7 years per HIPAA regulations. These documents should be retained for a longer period, however, if required by local regulations or institutional policies. No records will be destroyed without the written consent of the sponsor-investigator.

### **10.1.9 protocol deviations**

A protocol deviation is any noncompliance with the clinical trial protocol, GCP, or SOP requirements. The noncompliance may be either on the part of the participant, the investigator, or the study site staff. As a result of deviations, corrective actions are to be developed by the site and implemented promptly. These practices are consistent with ICH E6, sections:

- 4.5 Compliance with Protocol, sections 4.5.1, 4.5.2, and 4.5.3.
- 5.1 Quality Assurance and Quality Control, section 5.1.1.
- 5.20 Noncompliance, sections 5.20.1 and 5.20.2.

It is the responsibility of the study team to use continuous vigilance to identify and report deviations. All deviations must be addressed in study source documents, reported to the study PI. Protocol deviations must be sent to the local IRB per institutional guidelines. The site PI/ study staff is responsible for knowing and adhering to their IRB requirements. Further details about the handling of protocol deviations will be included in the -SOP and/or study procedures manual.

### **10.1.11 publication and data sharing policy**

This study will ensure that the public has access to the published results of this research.

### **10.1.12 conflict of interest policy**

Independence of this study from any actual or perceived influence, such as by the pharmaceutical industry, is critical. Any actual conflict of interest of persons who have a role in the design, conduct, analysis, publication, or any aspect of this trial will be disclosed and managed.by the University of Colorado Denver’s (UCD) Office of Regulatory Compliance Conflict of Interest and Commitment Management (COIC) program. Persons with a perceived conflict of interest will have such conflicts managed in a way that is appropriate to their participation in the trial. Conflict of Interest management plans are project-specific and are reviewed at least annually. UCD has integrated the institutional conflict of interest management program with its existing program.

# 11 references

1. King DK, Shoup JA, Raebel MA, et al. Planning for Implementation Success Using RE-AIM and CFIR Frameworks: A Qualitative Study. *Front Public Health*. 2020;8:59. doi:10.3389/fpubh.2020.00059

2. Weiner BJ, Lewis CC, Stanick C, et al. Psychometric assessment of three newly developed implementation outcome measures. *Implement Sci*. Aug 29 2017;12(1):108. doi:10.1186/s13012-017-0635-3

3. Laumann EO, Paik A, Rosen RC. Sexual dysfunction in the United States: prevalence and predictors. *JAMA*. Feb 10 1999;281(6):537-44.

4. van Dijk EM, van Dulmen-den Broeder E, Kaspers GJ, van Dam EW, Braam KI, Huisman J. Psychosexual functioning of childhood cancer survivors. *Psychooncology*. May 2008;17(5):506-11. doi:10.1002/pon.1274

5. Wettergren L, Kent EE, Mitchell SA, et al. Cancer negatively impacts on sexual function in adolescents and young adults: The AYA HOPE study. *Psychooncology*. Oct 2017;26(10):1632-1639. doi:10.1002/pon.4181

6. Zebrack BJ, Foley S, Wittmann D, Leonard M. Sexual functioning in young adult survivors of childhood cancer. *Psychooncology*. Aug 2010;19(8):814-22. doi:10.1002/pon.1641

7. Bober SL, Zhou ES, Chen B, Manley PE, Kenney LB, Recklitis CJ. Sexual function in childhood cancer survivors: a report from Project REACH. *J Sex Med*. Aug 2013;10(8):2084-93. doi:10.1111/jsm.12193

8. Haavisto A, Henriksson M, Heikkinen R, Puukko-Viertomies LR, Jahnukainen K. Sexual function in male long-term survivors of childhood acute lymphoblastic leukemia. *Cancer*. Jul 15 2016;122(14):2268-76. doi:10.1002/cncr.29989

9. Ritenour CW, Seidel KD, Leisenring W, et al. Erectile Dysfunction in Male Survivors of Childhood Cancer-A Report From the Childhood Cancer Survivor Study. *J Sex Med*. Jun 2016;13(6):945-54. doi:10.1016/j.jsxm.2016.03.367

10. Ford JS, Kawashima T, Whitton J, et al. Psychosexual functioning among adult female survivors of childhood cancer: a report from the childhood cancer survivor study. *J Clin Oncol*. Oct 1 2014;32(28):3126-36. doi:10.1200/JCO.2013.54.1086

11. van Iersel L, Li Z, Chemaitilly W, et al. Erectile Dysfunction in Male Survivors of Childhood Cancer. *JAMA Oncol*. Nov 1 2018;4(11):1613-1616. doi:10.1001/jamaoncol.2018.4420

12. Weinfurt KP, Lin L, Bruner DW, et al. Development and Initial Validation of the PROMIS((R)) Sexual Function and Satisfaction Measures Version 2.0. *J Sex Med*. Sep 2015;12(9):1961-74. doi:10.1111/jsm.12966

13. Bjornard KL, Howell CR, Klosky JL, et al. Psychosexual Functioning of Female Childhood Cancer Survivors: A Report From the St. Jude Lifetime Cohort Study. *J Sex Med*. Jul 25 2020;doi:10.1016/j.jsxm.2020.06.005

14. Sundberg KK, Lampic C, Arvidson J, Helstrom L, Wettergren L. Sexual function and experience among long-term survivors of childhood cancer. *Eur J Cancer*. Feb 2011;47(3):397-403. doi:10.1016/j.ejca.2010.09.040

15. Demedis J, Gupta A, Appiah LC, Chow EJ, Peterson PN. Sexual Dysfunction in Adolescent and Young Adult Survivors of Childhood Cancer: Presentation, Risk Factors, and Evaluation of an Underdiagnosed Late Effect: A Narrative Review. *J Adolesc Young Adult Oncol*. May 7 2020;doi:10.1089/jayao.2020.0025

16. Acquati C, Zebrack BJ, Faul AC, et al. Sexual functioning among young adult cancer patients: A 2-year longitudinal study. *Cancer*. Jan 15 2018;124(2):398-405. doi:10.1002/cncr.31030

17. Carpentier MY, Fortenberry JD. Romantic and sexual relationships, body image, and fertility in adolescent and young adult testicular cancer survivors: a review of the literature. *J Adolesc Health*. Aug 2010;47(2):115-25. doi:10.1016/j.jadohealth.2010.04.005

18. Children’s Oncology Group. Long-Term Follow Up Guidelines for Survivors of Childhood, Adolescent and Young Adult Cancer, Version 5.0. Children’s Oncology Group. Updated October. 2018. [www.survivorshipguidelines.org](file:///C:\Users\131208\AppData\Local\Microsoft\Windows\INetCache\Content.Outlook\HGMBUI1D\www.survivorshipguidelines.org)

19. Moroney MR, Flink D, Sheeder J, et al. Radiation therapy is not an independent risk factor for decreased sexual function in women with gynecologic cancers. *Rep Pract Oncol Radiother*. Sep-Oct 2018;23(5):331-336. doi:10.1016/j.rpor.2018.07.007

20. Guntupalli SR, Sheeder J, Ioffe Y, et al. Sexual and Marital Dysfunction in Women With Gynecologic Cancer. *Int J Gynecol Cancer*. Mar 2017;27(3):603-607. doi:10.1097/IGC.0000000000000906

21. Kenney LB, Cohen LE, Shnorhavorian M, et al. Male reproductive health after childhood, adolescent, and young adult cancers: a report from the Children's Oncology Group. *J Clin Oncol*. Sep 20 2012;30(27):3408-16. doi:10.1200/JCO.2011.38.6938

22. Kiserud CE, Schover LR, Dahl AA, et al. Do male lymphoma survivors have impaired sexual function? *J Clin Oncol*. Dec 10 2009;27(35):6019-26. doi:10.1200/JCO.2009.23.2280

23. Canada AL, Schover LR, Li Y. A pilot intervention to enhance psychosexual development in adolescents and young adults with cancer. *Pediatr Blood Cancer*. Nov 2007;49(6):824-8. doi:10.1002/pbc.21130

24. Bober SL, Recklitis CJ, Michaud AL, Wright AA. Improvement in sexual function after ovarian cancer: Effects of sexual therapy and rehabilitation after treatment for ovarian cancer. *Cancer*. Jan 1 2018;124(1):176-182. doi:10.1002/cncr.30976

25. Schover LR. Sexual quality of life in men and women after cancer. *Climacteric*. Oct 31 2018:1-5. doi:10.1080/13697137.2018.1526893

26. Frederick NN, Recklitis CJ, Blackmon JE, Bober S. Sexual Dysfunction in Young Adult Survivors of Childhood Cancer. *Pediatr Blood Cancer*. Sep 2016;63(9):1622-8. doi:10.1002/pbc.26041

27. Demedis J, Marsh R, Appiah LC, Klosky JL, Peterson PN, DorseyHolliman B. Evaluating sexual function in adolescent and young adult childhood cancer survivors. *Journal of Clinical Oncology*. 2020;38(15_suppl):e24180-e24180. doi:10.1200/JCO.2020.38.15_suppl.e24180

28. Krouwel EM, Albers LF, Nicolai MPJ, et al. Discussing Sexual Health in the Medical Oncologist's Practice: Exploring Current Practice and Challenges. *J Cancer Educ*. Jun 17 2019;doi:10.1007/s13187-019-01559-6

29. Park ER, Bober SL, Campbell EG, Recklitis CJ, Kutner JS, Diller L. General internist communication about sexual function with cancer survivors. *J Gen Intern Med*. Nov 2009;24 Suppl 2:S407-11. doi:10.1007/s11606-009-1026-5

30. Denlinger CS, Sanft T, Baker KS, et al. Survivorship, Version 2.2017, NCCN Clinical Practice Guidelines in Oncology. *J Natl Compr Canc Netw*. Sep 2017;15(9):1140-1163. doi:10.6004/jnccn.2017.0146

31. Carter J, Lacchetti C, Andersen BL, et al. Interventions to Address Sexual Problems in People With Cancer: American Society of Clinical Oncology Clinical Practice Guideline Adaptation of Cancer Care Ontario Guideline. *J Clin Oncol*. Feb 10 2018;36(5):492-511. doi:10.1200/JCO.2017.75.8995

32. Frederick NN, Revette A, Michaud A, Bober SL. A qualitative study of sexual and reproductive health communication with adolescent and young adult oncology patients. *Pediatr Blood Cancer*. Feb 15 2019:e27673. doi:10.1002/pbc.27673

33. Jervaeus A, Nilsson J, Eriksson LE, Lampic C, Widmark C, Wettergren L. Exploring childhood cancer survivors' views about sex and sexual experiences -findings from online focus group discussions. *Eur J Oncol Nurs*. Feb 2016;20:165-72. doi:10.1016/j.ejon.2015.07.009

34. Frederick NN, Campbell K, Kenney LB, Moss K, Speckhart A, Bober SL. Barriers and facilitators to sexual and reproductive health communication between pediatric oncology clinicians and adolescent and young adult patients: The clinician perspective. *Pediatr Blood Cancer*. Aug 2018;65(8):e27087. doi:10.1002/pbc.27087

35. Stinson JN, Jibb LA, Greenberg M, et al. A Qualitative Study of the Impact of Cancer on Romantic Relationships, Sexual Relationships, and Fertility: Perspectives of Canadian Adolescents and Parents During and After Treatment. *J Adolesc Young Adult Oncol*. Jun 2015;4(2):84-90. doi:10.1089/jayao.2014.0036

36. Chulani VL, Gordon LP. Adolescent growth and development. *Prim Care*. Sep 2014;41(3):465-87. doi:10.1016/j.pop.2014.05.002

37. Arnett JJ. Emerging adulthood: A theory of development from the late teens through the twenties. *American Psychologist*. 2000;55(5):469-480. doi:10.1037/0003-066x.55.5.469

38. Aubin S, Perez S. The Clinician's Toolbox: Assessing the Sexual Impacts of Cancer on Adolescents and Young Adults with Cancer (AYAC). *Sex Med*. Sep 2015;3(3):198-212. doi:10.1002/sm2.75

39. Flynn KE, Lin L, Cyranowski JM, et al. Development of the NIH PROMIS (R) Sexual Function and Satisfaction measures in patients with cancer. *J Sex Med*. Feb 2013;10 Suppl 1:43-52. doi:10.1111/j.1743-6109.2012.02995.x

40. Flynn KE, Reeve BB, Lin L, Cyranowski JM, Bruner DW, Weinfurt KP. Construct validity of the PROMIS(R) sexual function and satisfaction measures in patients with cancer. *Health Qual Life Outcomes*. Mar 11 2013;11:40. doi:10.1186/1477-7525-11-40

41. Carter J, Stabile C, Seidel B, Baser RE, Goldfarb S, Goldfrank DJ. Vaginal and sexual health treatment strategies within a female sexual medicine program for cancer patients and survivors. *J Cancer Surviv*. Apr 2017;11(2):274-283. doi:10.1007/s11764-016-0585-9

42. Demedis J, Marsh R, Ziniel SI, et al. Evaluation of the v2.0 Brief Profiles for Sexual Function and Satisfaction PROMIS in Adolescent and Young Adult Childhood Cancer Survivors. *J Adolesc Young Adult Oncol*. Nov 2 2020;doi:10.1089/jayao.2020.0166

43. Damschroder LJ, Reardon CM, AuYoung M, et al. Implementation findings from a hybrid III implementation-effectiveness trial of the Diabetes Prevention Program (DPP) in the Veterans Health Administration (VHA). *Implement Sci*. Jul 26 2017;12(1):94. doi:10.1186/s13012-017-0619-3

44. Arrossi S, Paolino M, Orellana L, Thouyaret L, Kohler RE, Viswanath K. Mixed-methods approach to evaluate an mHealth intervention to increase adherence to triage of human papillomavirus-positive women who have performed self-collection (the ATICA study): study protocol for a hybrid type I cluster randomized effectiveness-implementation trial. *Trials*. Feb 26 2019;20(1):148. doi:10.1186/s13063-019-3229-3

45. Moullin JC, Dickson KS, Stadnick NA, et al. Ten recommendations for using implementation frameworks in research and practice. *Implement Sci Commun*. 2020;1:42. doi:10.1186/s43058-020-00023-7

46. Glasgow RE, Harden SM, Gaglio B, et al. RE-AIM Planning and Evaluation Framework: Adapting to New Science and Practice With a 20-Year Review. *Front Public Health*. 2019;7:64. doi:10.3389/fpubh.2019.00064

47. Glasgow RE, Vogt TM, Boles SM. Evaluating the public health impact of health promotion interventions: the RE-AIM framework. *Am J Public Health*. Sep 1999;89(9):1322-7.

48. Damschroder LJ, Aron DC, Keith RE, Kirsh SR, Alexander JA, Lowery JC. Fostering implementation of health services research findings into practice: a consolidated framework for advancing implementation science. *Implement Sci*. Aug 7 2009;4:50. doi:10.1186/1748-5908-4-50

49. Keith RE, Crosson JC, O'Malley AS, Cromp D, Taylor EF. Using the Consolidated Framework for Implementation Research (CFIR) to produce actionable findings: a rapid-cycle evaluation approach to improving implementation. *Implement Sci*. Feb 10 2017;12(1):15. doi:10.1186/s13012-017-0550-7

50. Guest G, Bunce A, Johnson L. How Many Interviews Are Enough?:An Experiment with Data Saturation and Variability. *Field Methods*. 2006;18(1):59-82. doi:10.1177/1525822x05279903

51. Olagunju TO, Liu Y, Liang LJ, et al. Disparities in the survivorship experience among Latina survivors of breast cancer. *Cancer*. Jun 1 2018;124(11):2373-2380. doi:10.1002/cncr.31342

52. De Marchis EH, Hessler D, Fichtenberg C, et al. Part I: A Quantitative Study of Social Risk Screening Acceptability in Patients and Caregivers. *Am J Prev Med*. Dec 2019;57(6 Suppl 1):S25-S37. doi:10.1016/j.amepre.2019.07.010

53. Proctor E, Silmere H, Raghavan R, et al. Outcomes for implementation research: conceptual distinctions, measurement challenges, and research agenda. *Adm Policy Ment Health*. Mar 2011;38(2):65-76. doi:10.1007/s10488-010-0319-7

54. Waltz TJ, Powell BJ, Fernandez ME, Abadie B, Damschroder LJ. Choosing implementation strategies to address contextual barriers: diversity in recommendations and future directions. *Implement Sci*. Apr 29 2019;14(1):42. doi:10.1186/s13012-019-0892-4

55. Faulkner L. Beyond the five-user assumption: benefits of increased sample sizes in usability testing. *Behav Res Methods Instrum Comput*. Aug 2003;35(3):379-83. doi:10.3758/bf03195514
