# Supplementary material for: Testing effectiveness and implementation of a standardized approach to sexual dysfunction screening among adolescent and young adult-aged survivors of childhood cancer: A type I hybrid, mixed methods trial protocol
Source: PLoS One. 2024 Jul 22;19(7):e0305677. doi: 10.1371/journal.pone.0305677 (PMC11262696; doi:10.1371/journal.pone.0305677)
Supplement: S2 File — (DOCX) [file pone.0305677.s002.docx]

**S.2 Study Appendix: Example participant surveys and interview guides**

Appendix 1. Patient stakeholder survey of effectiveness and implementation (Aims 2-3)

Appendix 2. Example medical stakeholder survey of implementation outcomes (Aim 3)

Appendix 3. Example medical stakeholder interview guide for implementation outcomes (responsible providers) (Aim 3)

Appendix 4. Example brief interview guide (non-responsible providers) (Aim 3)

Appendix 5. Single item reach question for non-completion (Aim 3)

**Appendix 1. Aims 2-3 Patient Surveys**

**Patient Survey for Effectiveness (Aim 2)**

Please answer the following questions about yourself:

- 1. Gender identity
     1. Male
     2. Female
     3. Transgender Female/Male-to-Female
     4. Transgender Male/Female-to-Male
     5. Non-binary, neither exclusively man nor woman
     6. Other, specify: _______________
     7. Prefer not to answer
  2. What is your current age? __________
  3. Race (check all that apply)
     1. White
     2. Black or African American
     3. Asian
     4. Native American or Other Pacific Islander
     5. American Indian or Alaska Native
     6. Other, specify: ______________
     7. Prefer not to answer
  4. Ethnicity
     1. Hispanic or Latino
     2. Not Hispanic or Latino
     3. Prefer not to answer
  5. Which of these applies to you?
     1. I am receiving treatment to cure my cancer (such as chemotherapy, radiation, immunotherapy, or other treatment)
     2. I have completed cancer treatment and am in remission
     3. I currently have cancer but am not receiving treatment
     4. Other, specify: _______________

Please answer the following questions regarding your experiences and opinions about the oncology clinic’s approach to sexual healthcare. The oncology clinic recently started using a questionnaire about sexual function, or how sex is going for you. We want to know if that process is helpful. There are no right or wrong answers to these questions. Your honest responses will help us understand how well (or not well) we are doing. Please note, these questions are for research purposes only and will not be seen by your medical team. Please notify your medical team if there are resources that you need.

1. Did you complete a screening questionnaire about sexual function in the past 2 weeks? (Y/N/Unsure)
2. Not including the screening questionnaire you may have completed, did someone on the healthcare team (oncologist, nurse, or other clinician) talk to you or ask you about any concerns you may have about sexual function? (Y/N)
3. <If Yes to 2> Overall, on a scale of 0 to 10, with 0 being the worst and 10 being the best, how satisfied are you with the discussion with your clinician regarding sexual function?
4. Before any recent conversations about sexual health or function, did you feel that you needed education or resources about sexual function? (Y/N)
5. <If Yes to 4> Did you receive the education or resources related to sexual function that you needed? (Y/N)
   - 1. If Yes: What type of education/resources did you receive (check all that apply)?
        - Discussion with a clinician
        - Websites
        - Pamphlets
        - Other: ____________
6. Before any recent conversations about sexual health or function, did you feel that you needed any medical care (such as bloodwork or to see a specialist) for sexual function concerns? (Y/N)
7. <If Yes to 6> Did you receive the healthcare related to sexual function that you needed? (Y/N)
   - 1. If Yes: What type of medical care did you receive (check all that apply)?
        - Referral to another doctor (gynecology, urology, endocrinology, sexual function clinic, reproductive doctor, etc.)
        - Referral to psychologist or mental health provider
        - Prescription
        - Blood work
        - Other: ____________
     2. What additional resources do you need that you *did not* receive?
        - Education (Discussion with provider, website, pamphlet, etc.)
        - Referral to another doctor (gynecology, urology, endocrinology, sexual function clinic, reproductive doctor, etc.)
        - Referral to psychologist or mental health provider
        - Prescription
        - Blood work
        - Other: ____________
8. Overall, on a scale of 0 to 10, with 0 being the worst and 10 being the best, how satisfied are you with the sexual healthcare that you have received?

**Example Patient Survey for Implementation Outcomes (Aim 3, to be modified and re-submitted to IRB prior to use, after completion of Aim 1):**

**[**branching logic only for patients who answered *yes* to Question 1 above after implementation of the screening approach]

Which of these did you experience (Check all that apply)?

- 1. Your provider talked to you about the risks to sexual health and function.
  2. You were taken to a clinic room on your own (without anyone you came to clinic with).
  3. You were invited to complete a sexual function screening questionnaire.
  4. You were given the opportunity to complete the sexual function screening questionnaire on a digital device.
  5. You had a private place to complete the sexual function questionnaire.
  6. A clinician (doctor, nurse, nurse practitioner, or other provider) followed up with you about your answers on the sexual function screening questionnaire by doing any of the following things (*check all that apply):*
     1. Asking if you would like to talk about your results
     2. Reviewing any of your answers or asking you more questions about your sexual health/function
     3. Providing you with additional information about sexual health or function
     4. Ordering tests (bloodwork, etc.)
     5. Referring you to someone to help with your concerns (another doctor, psychology/therapy, physical therapy, etc.) to address your sexual health/function concerns

**For the items below, please mark your level of agreement with each statement.**

| **Acceptability of Intervention** | Not applicable | Completely disagree | Disagree | Neither agree nor disagree | Agree | Completely agree |
| --- | --- | --- | --- | --- | --- | --- |
| 1. I liked having the opportunity to answer questions about my sexual function. |  | ➀ | ➁ | ➂ | ➃ | ➄ |
| 2. I felt comfortable answering written sexual function questions. |  | ➀ | ➁ | ➂ | ➃ | ➄ |
| 3. I liked having oncology clinicians follow up with me about my responses to the sexual function questions. | 🄋 | ➀ | ➁ | ➂ | ➃ | ➄ |

**Intervention Appropriateness Measure (IAM)**

|  | Completely disagree | Disagree | Neither agree nor disagree | Agree | Completely agree |
| --- | --- | --- | --- | --- | --- |
| 1. I recommend this sexual function screening process should be available to other patients like me. | ➀ | ➁ | ➂ | ➃ | ➄ |
| 2. Sexual function screening is relevant to oncology patients my age. | ➀ | ➁ | ➂ | ➄ | ➄ |

**Feasibility of Intervention**

|  | Not applicable | Completely disagree | Disagree | Neither agree nor disagree | Agree | Completely agree |
| --- | --- | --- | --- | --- | --- | --- |
| 1. Completing the sexual function screening questionnaire in clinic was easy to do. |  | ➀ | ➁ | ➂ | ➃ | ➄ |
| 2. I was able to receive the care I needed regarding my sexual health or function. (with N/A option) | 🄋 | ➀ | ➁ | ➂ | ➃ | ➄ |
| 3. My privacy was protected during the sexual function while receiving sexual healthcare. |  | ➀ | ➁ | ➂ | ➃ | ➄ |

If you could anything about answering sexual function questionnaire, what would it be and why? ______________________________________________________________________________________________________________________________________________________________________________________________________________________________________________________________________________________________________

If you could anything about how your healthcare team followed up on your sexual function questionnaire (such as how they communicated about it, how they provided you with information or healthcare), what would it be and why? ______________________________________________________________________________________________________________________________________________________________________________________________________________________________________________________________________________________________________

**Appendix 2.**

**Example Provider Survey for Implementation Outcomes (Aim 3, to be modified and re-submitted to IRB prior to use, after completion of Aim 1):**

1. Which clinical group are you involved in (check all that apply):
   1. Liquid Tumor team
   2. Solid Tumor team
   3. Neuro-oncology team
   4. Survivorship
   5. None of the above. My clinical team is _____________
2. What is your role in the oncology clinic?
   1. Medical assistant
   2. RN/Nurse care coordinator
   3. Physician Assistant
   4. Nurse practitioner
   5. Fellow
   6. Attending
3. How long have you been in practice in your current role?
   1. 0-5 years
   2. 5-10 years
   3. More than 10 years

Your clinic recently implemented use of the PROMIS SexFS Brief, which is a short patient-reported outcome measure, for screening patients age 15-24 for sexual function concerns. The purpose of this screening is to address a patient-identified gap in the care provided by most oncologists. Your clinic also created a team of providers that helps to respond to the results of this questionnaire, and some provider resources. These questions are about how this process is working for you and your patients.

1. When you were the assigned sexual healthcare provider, how often did you review sexual function screening results for patients who had completed the questionnaire?
   1. Never
   2. Occasionally
   3. Some of the time
   4. Most of the time
   5. Every time
2. When you were the assigned sexual healthcare provider, how often did you discuss sexual function screening results with patients after they completed the questionnaire?
   1. Never
   2. Occasionally Some of the time
   3. Most of the time
   4. Every time

**For the items below, please mark your level of agreement with each statement. Questions will be divided into 3 focuses:**

1. **The overall sexual healthcare process (screening questionnaire and follow-up/management)**
2. **The screening/questionnaire process**
3. **The follow-up/management process**

**The below questions are about the OVERALL PROCESS of providing sexual function healthcare (*including both the use of the PROMIS SexFS Brief screening tool and the follow-up/management process*)**

*[Note: actual survey will not include survey labels, though each section will be on a different survey page]*

**Acceptability of Intervention Measure (AIM)**

|  | Completely disagree | Disagree | Neither agree nor disagree | Agree | Completely agree |
| --- | --- | --- | --- | --- | --- |
| 1. The current approach to sexual function healthcare meets my approval. | ➀ | ➁ | ➂ | ➃ | ➄ |
| 2. The current approach to sexual function healthcare is appealing to me. | ➀ | ➁ | ➂ | ➃ | ➄ |
| 3. I like the current approach to sexual function healthcare. | ➀ | ➁ | ➂ | ➃ | ➄ |
| 4. I welcome the current approach to sexual function healthcare. | ➀ | ➁ | ➂ | ➃ | ➄ |

**Intervention Appropriateness Measure (IAM)**

|  | Completely disagree | Disagree | Neither agree nor disagree | Agree | Completely agree |
| --- | --- | --- | --- | --- | --- |
| 1. The current approach to sexual function healthcare seems fitting. | ➀ | ➁ | ➂ | ➃ | ➄ |
| 2. The current approach to sexual function healthcare seems suitable. | ➀ | ➁ | ➂ | ➃ | ➄ |
| 3. The current approach to sexual function healthcare seems applicable to my patients and their needs. | ➀ | ➁ | ➂ | ➃ | ➄ |
| 4. The current approach to sexual function healthcare seems like a good match for my clinic. | ➀ | ➁ | ➂ | ➃ | ➄ |

**Feasibility of Intervention Measure (FIM)**

|  | Completely disagree | Disagree | Neither agree nor disagree | Agree | Completely agree |
| --- | --- | --- | --- | --- | --- |
| 1. The current approach to sexual function healthcare seems implementable. | ➀ | ➁ | ➂ | ➃ | ➄ |
| 2. Continuing the current approach to sexual function healthcare seems possible. | ➀ | ➁ | ➂ | ➃ | ➄ |
| 3. The current approach to sexual function healthcare seems doable. | ➀ | ➁ | ➂ | ➃ | ➄ |
| 4. The current approach to sexual function healthcare seems easy to use. | ➀ | ➁ | ➂ | ➃ | ➄ |

**Overall Impression**

1. Would you want your clinic to continue using this approach to sexual function healthcare? Y/N
2. Would you recommend this approach to sexual function healthcare process to similar clinics? Y/N

**The below questions are specifically about the process of having patients COMPLETE THE SEXUAL FUNCTION QUESTIONNAIRE.**

**Acceptability of Intervention Measure (AIM)**

|  | Completely disagree | Disagree | Neither agree nor disagree | Agree | Completely agree |
| --- | --- | --- | --- | --- | --- |
| 1. The current sexual function screening process meets my approval. | ➀ | ➁ | ➂ | ➃ | ➄ |
| 2. The current sexual function screening process is appealing to me. | ➀ | ➁ | ➂ | ➃ | ➄ |
| 3. I like the current sexual function screening process. | ➀ | ➁ | ➂ | ➃ | ➄ |
| 4. I welcome the current sexual function screening process. | ➀ | ➁ | ➂ | ➃ | ➄ |

**Intervention Appropriateness Measure (IAM)**

|  | Completely disagree | Disagree | Neither agree nor disagree | Agree | Completely agree |
| --- | --- | --- | --- | --- | --- |
| 1. The sexual function screening process seems fitting. | ➀ | ➁ | ➂ | ➃ | ➄ |
| 2. The sexual function screening process seems suitable. | ➀ | ➁ | ➂ | ➃ | ➄ |
| 3. The sexual function screening process seems applicable to my patients and their needs. | ➀ | ➁ | ➂ | ➃ | ➄ |
| 4. The sexual function screening process seems like a good match for my clinic. | ➀ | ➁ | ➂ | ➃ | ➄ |

**Feasibility of Intervention Measure (FIM)**

|  | Completely disagree | Disagree | Neither agree nor disagree | Agree | Completely agree |
| --- | --- | --- | --- | --- | --- |
| 1. The sexual function screening process seems implementable. | ➀ | ➁ | ➂ | ➃ | ➄ |
| 2. Continuing the sexual function screening process seems possible. | ➀ | ➁ | ➂ | ➃ | ➄ |
| 3. The sexual function screening process seems doable. | ➀ | ➁ | ➂ | ➃ | ➄ |
| 4. The sexual function screening process seems easy to use. | ➀ | ➁ | ➂ | ➃ | ➄ |

**Workflow and Resources**

|  | Completely disagree | Disagree | Neither agree nor disagree | Agree | Completely agree |
| --- | --- | --- | --- | --- | --- |
| 1. The workflow of patients answering sexual function questions is working well. | ➀ | ➁ | ➂ | ➃ | ➄ |

**The below questions are specifically about the process of providing FOLLOW-UP AND MANAGEMENT after patients have completed the sexual function questionnaire.**

**Acceptability of Intervention Measure (AIM)**

|  | Completely disagree | Disagree | Neither agree nor disagree | Agree | Completely agree |
| --- | --- | --- | --- | --- | --- |
| 1. The current sexual function management process meets my approval. | ➀ | ➁ | ➂ | ➃ | ➄ |
| 2. The current sexual function management process is appealing to me. | ➀ | ➁ | ➂ | ➃ | ➄ |
| 3. I like the current sexual function management process. | ➀ | ➁ | ➂ | ➃ | ➄ |
| 4. I welcome the current sexual function management process. | ➀ | ➁ | ➂ | ➃ | ➄ |

**Intervention Appropriateness Measure (IAM)**

|  | Completely disagree | Disagree | Neither agree nor disagree | Agree | Completely agree |
| --- | --- | --- | --- | --- | --- |
| 1. The sexual function management process seems fitting. | ➀ | ➁ | ➂ | ➃ | ➄ |
| 2. The sexual function management process seems suitable. | ➀ | ➁ | ➂ | ➃ | ➄ |
| 3. The sexual function management process seems applicable to my patients and their needs. | ➀ | ➁ | ➂ | ➃ | ➄ |
| 4. The sexual function management process seems like a good match for my clinic. | ➀ | ➁ | ➂ | ➃ | ➄ |

**Feasibility of Intervention Measure (FIM)**

|  | Completely disagree | Disagree | Neither agree nor disagree | Agree | Completely agree |
| --- | --- | --- | --- | --- | --- |
| 1. The sexual function management process seems implementable. | ➀ | ➁ | ➂ | ➃ | ➄ |
| 2. Continuing the sexual function management process seems possible. | ➀ | ➁ | ➂ | ➃ | ➄ |
| 3. The sexual function management process seems doable. | ➀ | ➁ | ➂ | ➃ | ➄ |
| 4. The sexual function management process seems easy to use. | ➀ | ➁ | ➂ | ➃ | ➄ |

**Workflow and Resources**

|  | Completely disagree | Disagree | Neither agree nor disagree | Agree | Completely agree |
| --- | --- | --- | --- | --- | --- |
| 1. The workflow of how screening results are addressed with patients is working well. | ➀ | ➁ | ➂ | ➃ | ➄ |
| 2. I have the resources (e.g. dedicated sexual health team members, written materials, etc.) that I need to address my patients concerns | ➀ | ➁ | ➂ | ➃ | ➄ |

**Appendix 3. Example medical stakeholder interview guide for implementation outcomes (responsible providers) (Aim 3, to be modified and re-submitted to IRB prior to use, after completion of adaptation aim):**

Thank you for speaking with me today. The interview will take roughly 30 minutes to an hour. Does this amount of time work for you?

First, I sent you an email with a link to a REDCap survey, which has the postcard consent form. If you haven’t already, please follow that link and read the consent statement. If you still agree, please click “I consent”. Please let me know if you have any questions.

Now, there are a few quick questions I’ll ask you to fill out on the same page. Please let me know once you are done with those.

1. Are you directly involved in patient screening or follow-up with the PROMIS SexFS Brief sexual function tool? Y/N
2. Which clinical group are you involved in (check all that apply):
   1. Liquid Tumor team
   2. Solid Tumor team
   3. Neuro-oncology team
   4. Survivorship
   5. None of the above. My clinical team is _____________
3. What is your role in the oncology clinic?
   1. Medical assistant
   2. RN/Nurse care coordinator
   3. Physician Assistant
   4. Nurse practitioner
   5. Fellow
   6. Attending
4. How long have you been in practice in your current role?
   1. 0-5 years
   2. 5-10 years
   3. More than 10 years

<End REDCap Survey>

Thank you so much. For the rest of our time, I will ask you a few questions about your experiences with the sexual function screening process that your clinic has been using with AYA patients, using the PROMIS SexFS Brief. To jog your memory, you answered some questions in an online survey recently about how feasible, appropriate, and acceptable the screening process was.

There are no right or wrong answers to these questions. We are seeking feedback from individuals in the clinic about their perceptions of the screening process and will learn from what you share with us, whether positive, negative, or neutral. Do you have any questions for me before we begin?

1. **First, can you talk about your general impression of this approach to sexual function screening?**

*Specific probes for use if participants do not address these issues spontaneously:*

- What do you know about the screening process or its implementation?
  - What has been your role during planning or implementation of the screening process?
- What do you think about this screening approach? How has it been going?
- What do you like about this screening approach?
- What do you dislike about this screening approach?
- How is this screening process relevant (or not relevant) to the needs of your AYA oncology patients?
  - Do others see a need for the intervention?
- How does the PROMIS SexFS Brief questionnaire compare to other approaches for screening AYA survivors for sexual dysfunction that you are aware of? Are there better alternatives?
  - If there is another approach mentioned: Can you describe how that would work in more detail? Why would that be preferred?

1. **Next, I’d like to review your survey responses.**

*Specific probes for use if participants do not address these issues spontaneously:*

- - On the survey about acceptability of this screening approach, you generally felt it was/was not acceptable *OR* had some missed responses (*interviewer* *provides examples*). Can you tell me more about why you answered that way?
  - On the survey about feasibility of this screening approach, you generally felt it was/was not feasible *OR* had some missed responses (*interviewer* *provides examples*). Can you tell me more about why you answered that way?
  - On the survey about appropriateness of this screening approach, you generally felt it was/was not an appropriate approach to address sexual function for your patients *OR* had some missed responses (*interviewer* *provides examples*). Can you tell me more about why you answered that way?

1. **I know that you previously said that this screening approach is going ______. Thinking specifically about existing work flow in your clinic, how well does the screening process fit?**

*Specific probes for use if participants do not address these issues spontaneously:*

- - Since this intervention was implemented in your clinic, what changes have been made to the screening approach? (or more effectively if already effective?)
  - Are there (additional) changes that could be made to make this intervention fit better in your clinic?
  - Are there components that should not be altered?

1. **Now, thinking about follow-up/results management, how is this process fitting within the existing work flow in your clinic?**
   - Since this intervention was implemented, what changes have been made to the follow-up or results management processes?
   - What (additional) changes could be made to make this intervention work more effectively in your clinic?
   - Are there aspects of the follow-up processes and results management that should not be altered?
2. **Can you tell me about general buy-in from your team for this sexual function screening process?**

*Specific probes for use if participants do not address these issues spontaneously:*

- - To what extent are staff aware of patients’ needs with regard to sexual function/health concerns?
  - Do others see a need for this screening intervention? What, if anything, helped this intervention get buy-in from others in your clinic?
  - What has been the role of leadership with regard to implementing the screening approach?
    1. What level of endorsement or support have you seen or heard from leaders on your team or in your unit?
    2. Who have these leaders been?
  - How confident do you and your colleagues feel about using the screening approach? What about in responding to patient concerns?

1. **What do you think the patient experience has been with this sexual function screening approach?**

*Specific probes for use if participants do not address these issues spontaneously:*

- - Have you heard anything about the experiences of patients with the intervention?
    1. Can you describe a specific story?
  - How well do you think the screening approach meets the needs of AYA oncology patients served by your organization?
  - In what ways does it meet their needs? (only offer if few or no responses: e.g. improved access to services? Reduced wait times? Help with self-management?)
  - What barriers do you think your clinic’s patients/survivors face that may limit their participation in this intervention?
  - Do you foresee any future barriers that may prevent individuals participating in this intervention the screening process?

1. **Let’s talk about resources that make it possible to continue to use this screening approach.**

*Specific probes for use if participants do not address these issues spontaneously:*

- - What kinds of resources have already been made available to you?
    1. Copies of materials?
    2. Internal information sharing or education; e.g., staff meetings?
    3. Clinical champions or other people you can go to for help
    4. Referral/management recommendations
  - Have these resources been timely? Relevant? Sufficient?
  - Are there resources that are not helpful or are not needed? Tell me more.
  - Who do you ask/would you ask if you have questions about the intervention or its implementation?
    1. How available are these individuals?
  - Are there additional resources that are necessary to support your clinic in using the screening approach going forward?

1. **I have a few more questions about the intervention as a whole.** What were some barriers or challenges you ran into when implementing this screening approach? What did you do about these?
   - What really helped to facilitate successful implementation of this sexual function screening approach?
   - Who should be involved in adapting or sustaining this sexual function screening approach?

**Appendix 4. Example brief interview guide (non-responsible providers) (Aim 3 to be modified and re-submitted to IRB prior to use, after completion of adaptation aim)**

Thank you for speaking with me today. The interview will take roughly 30 minutes to an hour. Does this amount of time work for you?

First, I sent you an email with a link to a REDCap survey, which has the postcard consent form. If you haven’t already, please follow that link and read the consent statement. If you still agree, please click “I consent”. Please let me know if you have any questions.

Now, there are few quick questions I’ll ask you to complete using the same link. Please let me know once you are done with those.

1. Which clinical group are you involved in (check all that apply):
   1. Liquid Tumor team
   2. Solid Tumor team
   3. Neuro-oncology team
   4. Survivorship
   5. None of the above. My clinical team is _____________
2. What is your role in the oncology clinic?
   1. Medical assistant
   2. RN/Nurse care coordinator
   3. Physician Assistant
   4. Nurse practitioner
   5. Fellow
   6. Attending
3. How long have you been in practice in your current role?
   1. 0-5 years
   2. 5-10 years
   3. More than 10 years

Thank you so much. This focus group is to review the current iteration of the clinic’s routine approach to sexual function screening in AYA patients using the PROMIS SexFS Brief. After receiving feedback from patients and providers through surveys and interviews, we made some adaptations to the screening approach. To jog your memory, I will review the screening approach and the changes that have been made to it thus far.

In this focus group, we want to make sure there is nothing that we overlooked before we roll this out clinic-wide. Do you have any questions for me before we begin?

1. **First, can you talk about your general impression of this approach to sexual function screening?**

*Specific probes for use if participants do not address these issues spontaneously:*

- What do you know about the screening process or its implementation?
  - What has been your role during planning or implementation of the screening process?
- What do you think about the screening approach? How has it been going?
- What do you like about this screening approach?
- What do you dislike about this screening approach?
- How is this screening process relevant (or not relevant) to the needs of your AYA oncology patients?
  - Do others see a need for the intervention?
- How does the PROMIS SexFS Brief questionnaire compare to other approaches for screening AYA survivors for sexual dysfunction? Are there better alternatives?
  - If there is another approach mentioned: Can you describe how that would work in more detail? Why would that be preferred?

1. **I know that you previously said that this screening approach is going ______. Thinking specifically about existing work flow in your clinic, how well does the current screening process fit?**

*Specific probes for use if participants do not address these issues spontaneously:*

- What kinds of changes or alterations do you need to make this screening process fit better in your setting? (or more effectively if already effective?)
- Are there parts of the screening approach that should not be altered? Why?

1. **Now, thinking about follow-up/results management, how is this process fitting within the existing work flow in your clinic?**
   - What kinds of changes would make the follow-up or results management processes fit better in your clinic (or more effectively if already effective?)?
   - Are there aspects of the follow-up processes and results management that should not be altered?
2. **Can you tell me about general buy-in from your team for this sexual function screening process?**

*Specific probes for use if participants do not address these issues spontaneously:*

- - To what extent are staff aware of patients’ needs with regard to sexual function/health concerns?
  - Do others see a need for the screening intervention?
  - What has been the role of leadership with regard to implementing the screening approach?
    1. What level of endorsement or support have you seen or heard from leaders on your team or in your unit?
    2. Who have these leaders been?
  - How do people feel about current programs/practices/process that are available related to the intervention?

1. **How do you think the patient experience has been with this sexual function screening approach?**

*Specific probes for use if participants do not address these issues spontaneously:*

- - Have you heard anything about the experiences of patients with the intervention?
    1. Can you describe a specific story?
  - How well do you think the screening approach meets the needs of AYA oncology patients served by your organization?
  - In what ways does it meet their needs? (only offer if few or no responses: e.g. improved access to services? Reduced wait times? Help with self-management?)
  - What barriers do you think your clinic’s patients/survivors face that may limit their participation in this intervention?
  - Do you foresee any future barriers that may prevent individuals participating in this intervention in the screening process?

1. **Let’s talk about resources for making it possible to continue to use this screening approach.**

*Specific probes for use if participants do not address these issues spontaneously:*

- - What kinds of resources have already been made available to you?
    1. Copies of materials?
    2. Internal information sharing or education; e.g., staff meetings?
    3. Clinical champions or other people you can go to for help
    4. Referral/management recommendations
    5. Has it been timely? Relevant? Sufficient?
  - How confident do you and your colleagues feel about using the screening approach? What about in responding to patient concerns?
  - Who do you ask if you have questions about the intervention or its implementation?
    1. How available are these individuals?
  - Are there additional resources that would help with continuing this screening approach in your clinic?

**Appendix 5. Single item reach question for non-completion (Aim 3)**

As part of our study, we are tracking reasons that patients do not complete the sexual function screening process. A patient you saw in clinic (*provide patient name and clinic appointment information)* was due for sexual function screening, but did not receive it within a month of it being due.

To the best of your knowledge, please select the reasons that this patient did not complete the sexual function screening (check all that apply):

- Screening was unintentionally not offered (i.e., mistakenly missed)
- Patient chose not to answer questions, including question about opting out of survey
- Parent choice/refusal
- Clinically inappropriate
  - Please describe why:____________
- Provider choice
  - Please describe why:____________
- Other: _________________
