# Supplementary material for: Testing effectiveness and implementation of a standardized approach to sexual dysfunction screening among adolescent and young adult-aged survivors of childhood cancer: A type I hybrid, mixed methods trial protocol
Source: PLoS One. 2024 Jul 22;19(7):e0305677. doi: 10.1371/journal.pone.0305677 (PMC11262696; doi:10.1371/journal.pone.0305677)
Supplement: S3 File — (PDF) [file pone.0305677.s003.pdf]

Affix label here

Principal Investigator: Jenna Demedis, MD, MS
COMIRB No: 22-0709
Version Date: January 5, 2024

Study Title: Stakeholder-Engaged Development and Evaluation of a Screening Approach for Sexual Dysfunction in Adolescent and Young Adult Patients with and Surviving Childhood Cancer

Assent Form: Minor Patient Assent

You are being asked to be in a research study. This form provides you with information about the study. A member of the research team will describe this study to you and answer all of your questions. Please read the information below and ask questions about anything you don't understand before deciding whether or not to take part.

Why is this study being done?

This study is being done to learn more about patients' experiences with how we ask about sexual health and function in our oncology clinics. You are being asked to participate because you are between the ages of 15 and 17 and have been diagnosed with cancer or have a history of cancer. If you agree to be in the study you will be one of up to 120 people who are being studied.

What happens if I join this study?

If you join the study, you will participate in a survey that will take approximately 5-10 minutes. This survey will ask you about whether or not your doctor or nurse spoke with you about sexual health and function during your regular oncology clinic visit. It will ask questions about whether or not you completed a screening questionnaire about this, and whether that process was for you, and if it was helpful.

Some participants will also be asked to take a second survey right after the first one. This depends on what portion of the research study that we are in.

In this study, we will also review your medical record and collect some data, such as any medical diagnoses you may have had, treatments used to treat your cancer, and if your doctor refers you to any other specialist doctors.

What are the possible discomforts or risks?

Possible discomforts or risks include a chance that people will feel uncomfortable answering questions about whether their doctor talked to them about sexual health. There may be risks the researchers have not thought of. Every effort will be made to protect your privacy and confidentiality, but there are some things we cannot keep private.

What are the possible benefits of the study?

This study is designed for the researcher to learn more if our clinic's approach to screening for sexual health and function concerns is helpful. What we learn from this study may help us create new programs to help future children with cancer. It will not provide any direct benefits to you.

Who is paying for this study?

This research is being paid for by National Institute of Health.

Will I be paid for being in the study?

You will be paid $10 for each survey. Participants who are completing 2 surveys will receive another $10 for a total of $20 for participating. The number of surveys you will complete and amount you will receive depends on which part of the study we are in. You will receive the $10 or $20 in the form of an Amazon® gift card.

Will I have to pay for anything?

It will not cost you anything to be in the study.

Is my participation voluntary?

Taking part in this study is voluntary. You have the right to choose not to take part in this study. If you choose to take part, you have the right to stop at any time. If you refuse or decide to withdraw later, you will not lose any benefits or rights to which you are entitled.

Can I be removed from this study?

The study doctor may decide to stop your participation without your permission if the study doctor thinks that being in the study may cause you harm, or for any other reason. Also, the sponsor may stop the study at any time.

What happens if I am injured or hurt during the study?

Children's Hospital Colorado has no plan to pay for any physical or psychological injury. If you are injured or hurt during this study, you may call Dr. Jenna Demedis at 720-777-0188. She will help you obtain the resources you need for any psychological injury.

Who do I call if I have questions?

The researcher carrying out this study is Dr. Jenna Demedis. You may ask any questions you have now. If you have questions later, you may call Dr. Jenna Demedis at 720-777-0188 or email her at jenna.demedis@cuanschutz.edu

You may have questions about your rights as someone in this study. You can call or email Dr. Jenna Demedis with questions. You can also call the Colorado Multiple Institutional Review Board (COMIRB). You can call them at 303-724-1055.

Things That Must be Reported to the Authorities

We respect your right to privacy. But there are some things we cannot keep private. If you give us information about child neglect or child abuse, we have to report that to Social Services. If you give us information about someone hurting someone else, we have to report that to the police. If a court orders us to hand over your study records, we have to hand them over to the court.

Who will see my research information?

We will do everything we can to keep your records a secret. It cannot be guaranteed.

Both the records that identify you and the assent form signed by you may be looked at by others.

- Federal agencies that monitor human subject research
- Human Subject Research Committee
- The group doing the study
- The group paying for the study
- Regulatory officials from the institution where the research is being conducted who want to make sure the research is safe

The data we collect will be used for this study but may also be important for future research. Your data may be used for future research or distributed to other researchers for future study without additional assent if information that identifies you is removed from the data.

The results from the research may be shared at a meeting. The results from the research may be in published articles. Your name will be kept private when information is presented.

Agreement to be in this study and use my data

I have read this paper about the study or it was read to me. I understand the possible risks and benefits of this study. I know that being in this study is voluntary. I choose to be in this study: I will get a signed and dated copy of this assent form.

Name of Subject:_______________________________________

Signature:____ Date:_____________

Print Name:____

Assent form explained by:______________________________ Date:_____________

Print Name: ____

Affix label here

Principal Investigator: Jenna Demedis, MD, MS
COMIRB No: 22-0709
Version Date: March 31, 2023

Study Title: Stakeholder-Engaged Development and Evaluation of a Screening Approach for Sexual Dysfunction in Adolescent and Young Adult Patients with and Surviving Childhood Cancer

Consent Form: Provider Focus Group Consent

You are being asked to be in a research study. This form provides you with information about the study. Please read the information below and ask questions about anything you don't understand before deciding whether or not to take part.

This study is being done to learn more about providers' and patients' experiences with how we ask about sexual health and function in our oncology clinics. You are being asked to participate because you are a medical provider caring for patients with or surviving cancer. If you agree to be in the study you will be one of up to 6 people who are participating.

If you join the study, you will participate in a videoconference focus group that will take approximately 60 minutes. This focus group will be with other medical providers to discuss your opinion of the most recent version of sexual function screening approach in your oncology clinic. We will also ask you some questions about you and your role in clinic.

Possible discomforts or risks include a chance that people will feel uncomfortable answering questions about their clinical practice with their patients. There may be risks the researchers have not thought of. Every effort will be made to protect your privacy and confidentiality, but there are some things we cannot keep private.

This study is not designed to benefit you directly.

This research is being paid for by National Institute of Health.

You will be paid $40 for participating. You will receive the $40 in the form of an Amazon® gift card.

You have a choice about being in this study. You do not have to be in this study if you do not want to be.

The data we collect will be used for this study but may also be important for future research. Your data may be used for future research or distributed to other researchers for future study without additional consent if information that identifies you is removed from the data.

This study has been issued a Certificate of Confidentiality from the federal government to help protect your privacy. The Certificate prohibits the researchers from disclosing your name, or any identifiable information, document or biospecimen from the research, with the exceptions listed

below. A certificate provides protections against disclosing research information in federal, state, or local civil, criminal, administrative, legislative or other proceedings.

These protections apply only to your research records. The protections do not apply to your medical records.

The researchers may disclose your name or identifiable information, document or biospecimen, under the following circumstances: To those connected with the research, if required by Federal, State or local laws, if necessary for your medical treatment, with your consent, for other scientific research conducted in compliance with Federal regulations, to comply with mandated reporting, such as a possible threat to harm yourself or others, reports of child abuse, and required communicable disease reporting, or under other circumstances with your consent.

A Certificate of Confidentiality does not protect information you or a member of your family voluntarily release.

If you have questions, you can call Jenna Demedis at 720-777-0188 or email her (jenna.demedis@cuanschutz.edu). You can call to ask questions at any time.

You may have questions about your rights as someone in this study. If you have questions, you can call COMIRB (the responsible Institutional Review Board) at (303) 724-1055.

By completing this survey, you are agreeing to participate in this research study.

Affix label here

Principal Investigator: Jenna Demedis, MD, MS
COMIRB No: 22-0709
Version Date: March 31, 2023

Study Title: Stakeholder-Engaged Development and Evaluation of a Screening Approach for Sexual Dysfunction in Adolescent and Young Adult Patients with and Surviving Childhood Cancer

Consent Form: Provider Interview Consent

You are being asked to be in a research study. This form provides you with information about the study. Please read the information below and ask questions about anything you don't understand before deciding whether or not to take part.

This study is being done to learn more about providers' and patients' experiences with how we ask about sexual health and function in our oncology clinics. You are being asked to participate because you are a medical provider caring for patients with or surviving cancer. If you agree to be in the study you will be one of up to 25 people who are participating.

If you join the study, you will participate in a phone interview that will take approximately 30-60 minutes. This interview will ask you your opinions of how screening for sexual function in your oncology clinic is going. We will also ask you some questions about you and your role in clinic. The interview will be digitally recorded.

Possible discomforts or risks include a chance that people will feel uncomfortable answering questions about their clinical practice with their patients. There may be risks the researchers have not thought of. Every effort will be made to protect your privacy and confidentiality, but there are some things we cannot keep private.

This study is not designed to benefit you directly.

This research is being paid for by National Institute of Health.

You will be paid $40 for participating. You will receive the $40 in the form of an Amazon® gift card.

You have a choice about being in this study. You do not have to be in this study if you do not want to be.

The data we collect will be used for this study but may also be important for future research. Your data may be used for future research or distributed to other researchers for future study without additional consent if information that identifies you is removed from the data.

This study has been issued a Certificate of Confidentiality from the federal government to help protect your privacy. The Certificate prohibits the researchers from disclosing your name, or any identifiable information, document or biospecimen from the research, with the exceptions listed

below. A certificate provides protections against disclosing research information in federal, state, or local civil, criminal, administrative, legislative or other proceedings.

These protections apply only to your research records. The protections do not apply to your medical records.

The researchers may disclose your name or identifiable information, document or biospecimen, under the following circumstances: To those connected with the research, if required by Federal, State or local laws, if necessary for your medical treatment, with your consent, for other scientific research conducted in compliance with Federal regulations, to comply with mandated reporting, such as a possible threat to harm yourself or others, reports of child abuse, and required communicable disease reporting, or under other circumstances with your consent.

A Certificate of Confidentiality does not protect information you or a member of your family voluntarily release.

If you have questions, you can call Jenna Demedis at 720-777-0188 or email her (jenna.Demedis@cuanschutz.edu). You can call to ask questions at any time.

You may have questions about your rights as someone in this study. If you have questions, you can call COMIRB (the responsible Institutional Review Board) at (303) 724-1055.

By completing this survey, you are agreeing to participate in this research study.

COMIRB
APPROVED
For Use
31-Mar-2023
30-Mar-2024

Affix label here

Principal Investigator: Jenna Demedis, MD, MS
COMIRB No: 22-0709
Version Date: March 31, 2023

Study Title: Stakeholder-Engaged Development and Evaluation of a Screening Approach for Sexual Dysfunction in Adolescent and Young Adult Patients with and Surviving Childhood Cancer

Consent Form: Provider Survey Consent

You are being asked to be in a research study. This form provides you with information about the study. Please read the information below and ask questions about anything you don't understand before deciding whether or not to take part.

This study is being done to learn more about providers' and patients' experiences with how we ask about sexual health and function in our oncology clinics. You are being asked to participate because you are a medical provider caring for patients with or surviving cancer. If you agree to be in the study you will be one of up to 50 people who are participating.

If you join the study, you will participate in a survey that will take approximately 5-10 minutes. This survey will ask you your opinions of how screening for sexual function in your oncology clinic is going. It will also ask you some questions about you and your role in clinic.

Possible discomforts or risks include a chance that people will feel uncomfortable answering questions about their clinical practice with their patients. There may be risks the researchers have not thought of. Every effort will be made to protect your privacy and confidentiality, but there are some things we cannot keep private.

This study is not designed to benefit you directly.

This research is being paid for by National Institute of Health.

You will be paid $10 for participating. You will receive the $10 in the form of an Amazon® gift card.

You have a choice about being in this study. You do not have to be in this study if you do not want to be.

The data we collect will be used for this study but may also be important for future research. Your data may be used for future research or distributed to other researchers for future study without additional consent if information that identifies you is removed from the data.

This study has been issued a Certificate of Confidentiality from the federal government to help protect your privacy. The Certificate prohibits the researchers from disclosing your name, or any identifiable information, document or biospecimen from the research, with the exceptions listed

below. A certificate provides protections against disclosing research information in federal, state, or local civil, criminal, administrative, legislative or other proceedings.

These protections apply only to your research records. The protections do not apply to your medical records.

The researchers may disclose your name or identifiable information, document or biospecimen, under the following circumstances: To those connected with the research, if required by Federal, State or local laws, if necessary for your medical treatment, with your consent, for other scientific research conducted in compliance with Federal regulations, to comply with mandated reporting, such as a possible threat to harm yourself or others, reports of child abuse, and required communicable disease reporting, or under other circumstances with your consent.

A Certificate of Confidentiality does not protect information you or a member of your family voluntarily release.

If you have questions, you can call Jenna Demedis at 720-777-0188 or email her (jenna.Demedis@cuanschutz.edu). You can call to ask questions at any time.

You may have questions about your rights as someone in this study. If you have questions, you can call COMIRB (the responsible Institutional Review Board) at (303) 724-1055.

By completing this survey, you are agreeing to participate in this research study.

Affix label here

Principal Investigator: Jenna Demedis, MD, MS
COMIRB No: 22-0709
Version Date: January 5, 2024

Study Title: Stakeholder-Engaged Development and Evaluation of a Screening Approach for Sexual Dysfunction in Adolescent and Young Adult Patients with and Surviving Childhood Cancer

Consent Form: Adult Patient Consent

You are being asked to be in a research study. This form provides you with information about the study. A member of the research team will describe this study to you and answer all of your questions. Please read the information below and ask questions about anything you don't understand before deciding whether or not to take part.

Why is this study being done?

This study is being done to learn more about patients' experiences with how we ask about sexual health and function in our oncology clinics. You are being asked to participate because you are between the ages of 18 and 24 and have been diagnosed with cancer or have a history of cancer. If you agree to be in the study you will be one of up to 120 people who are being studied.

What happens if I join this study?

If you join the study, you will participate in a survey that will take approximately 5-10 minutes. This survey will ask you about whether or not your doctor or nurse spoke with you about sexual health and function during your regular oncology clinic visit. It will ask questions about whether or not you completed a screening questionnaire about this, and whether that process was for you, and if it was helpful.

Some participants will also be asked to take a second survey right after the first one. This depends on what portion of the research study that we are in.

In this study, we will also review your medical record and collect some data, such as any medical diagnoses you may have had, treatments used to treat your cancer, and if your doctor refers you to any other specialist doctors.

What are the possible discomforts or risks?

Possible discomforts or risks include a chance that people will feel uncomfortable answering questions about whether their doctor talked to them about sexual health. There may be risks the researchers have not thought of. Every effort will be made to protect your privacy and confidentiality, but there are some things we cannot keep private.

What are the possible benefits of the study?

This study is designed for the researcher to learn more if our clinic's approach to screening for sexual health and function concerns is helpful. What we learn from this study may help us create new programs to help future children with cancer. It will not provide any direct benefits to you.

Who is paying for this study?

This research is being paid for by National Institute of Health.

Will I be paid for being in the study?

You will be paid $10 for each survey. Participants who are completing 2 surveys will receive another $10 for a total of $20 for participating. The number of surveys you will complete and amount you will receive depends on which part of the study we are in. You will receive the $10 or $20 in the form of an Amazon® gift card.

Will I have to pay for anything?

It will not cost you anything to be in the study.

Is my participation voluntary?

Taking part in this study is voluntary. You have the right to choose not to take part in this study. If you choose to take part, you have the right to stop at any time. If you refuse or decide to withdraw later, you will not lose any benefits or rights to which you are entitled.

Can I be removed from this study?

The study doctor may decide to stop your participation without your permission if the study doctor thinks that being in the study may cause you harm, or for any other reason. Also, the sponsor may stop the study at any time.

What happens if I am injured or hurt during the study?

Children's Hospital Colorado has no plan to pay for any physical or psychological injury. If you are injured or hurt during this study, you may call Dr. Jenna Demedis at 720-777-0188. She will help you obtain the resources you need for any psychological injury.

Who do I call if I have questions?

The researcher carrying out this study is Dr. Jenna Demedis. You may ask any questions you have now. If you have questions later, you may call Dr. Jenna Demedis at 720-777-0188 or email her at jenna.Demedis@cuanschutz.edu

You may have questions about your rights as someone in this study. You can call or email Dr. Jenna Demedis with questions. You can also call the Colorado Multiple Institutional Review Board (COMIRB). You can call them at 303-724-1055.

Things That Must be Reported to the Authorities

We respect your right to privacy. But there are some things we cannot keep private. If you give us information about child neglect or child abuse, we have to report that to Social Services. If you give us information about someone hurting someone else, we have to report that to the police. If a court orders us to hand over your study records, we have to hand them over to the court.

Certificate of Confidentiality

This study has been issued a Certificate of Confidentiality from the federal government to help protect your privacy. The Certificate prohibits the researchers from disclosing your name, or any identifiable information, document or biospecimen from the research, with the exceptions listed below. A certificate provides protections against disclosing research information in federal, state, or local civil, criminal, administrative, legislative or other proceedings.

These protections apply only to your research records. The protections do not apply to your medical records.

The researchers may disclose your name or identifiable information, document or biospecimen, under the following circumstances:

- To those connected with the research,
- If required by Federal, State or local laws,
- If necessary for your medical treatment, with your consent,
- For other scientific research conducted in compliance with Federal regulations,
- To comply with mandated reporting, such as a possible threat to harm yourself or others, reports of child abuse, and required communicable disease reporting, or
- Under other circumstances with your consent.

A Certificate of Confidentiality does not protect information you or a member of your family voluntarily release.

Who will see my research information?

The University of Colorado Denver | Anschutz Medical Campus and its affiliated hospital(s) have rules to protect information about you. Federal and state laws including the Health Insurance Portability and Accountability Act (HIPAA) also protect your privacy. This part of the consent form tells you what information about you may be collected in this study and who might see or use it.

The institutions involved in this study include:

- Children's Hospital Colorado (Children's Colorado)
- University of Colorado Denver (UCD) | Anschutz Medical Campus

Children's Colorado shares a medical record system with the Barbara Davis Center and PedsConnect; therefore, it is also possible that other healthcare professionals could view your information.

We cannot do this study without your permission to see, use and give out your information. You do not have to give us this permission. If you do not, then you may not join this study.

We will see, use and disclose your information only as described in this form and in our Notice of Privacy Practices; however, people outside the UCD and its affiliate hospitals may not be covered by this obligation.

We will do everything we can to maintain the confidentiality of your personal information but confidentiality cannot be guaranteed.

The use and disclosure of your information has no time limit. You can cancel your permission to use and disclose your information at any time by writing to the study's Principal Investigator (PI), at the name and address listed below. If you do cancel your permission to use and disclose your information, your part in this study will end and no further information about you will be collected. Your cancellation would not affect information already collected in this study.

Jenna Demedis, MD, MS
Children's Hospital Colorado
13123 East 16th Avenue, B115
Aurora, CO 80045

Both the research records that identify you and the consent form signed by you may be looked at by others who have a legal right to see that information.

- Federal offices such as the Food and Drug Administration (FDA) and the Office of Human Research Protections (OHRP) that protect research subjects like you.
- People at the Colorado Multiple Institutional Review Board (COMIRB)
- The study doctor and the rest of the study team.
- Officials at the institution where the research is being conducted and officials at other institutions involved in this study who are in charge of making sure that we follow all of the rules for research
- National Institutes of Health (NIH), the group paying for this research study

We might talk about this research study at meetings. We might also print the results of this research study in relevant journals. But we will always keep the names of the research subjects, like you, private.

You have the right to request access to your personal health information from the Investigator.

Information about you that will be seen, collected, used and disclosed in this study:

- Name and Demographic Information (age, sex, ethnicity, address, phone number, etc.)
- Medical Record Number
- Portions of my previous and current Medical Records that are relevant to this study, including but not limited to Diagnosis(es), History and Physical, laboratory or tissue studies, radiology studies, procedure results, referral orders
- Research Visit and Research Test records
- Financial and Billing Information

What happens to data that are collected in this study?

Scientists at the University and the health systems involved in this study work to improve healthcare. The data collected from you during this study are important to this study and to future research. If you join this study:

- The data are given by you to the investigators for this research and so no longer belong to you.
- Both the investigators and any sponsor of this research may study your data collected from you.
- If data are in a form that identifies you, the University or the health systems involved in this study may use them for future research only with your consent or IRB approval.
- Any product or idea created by the researchers working on this study will not belong to you.
- There is no plan for you to receive any financial benefit from the creation, use or sale of such a product or idea.

Agreement to be in this study and use my data

I have read this paper about the study or it was read to me. I understand the possible risks and benefits of this study. I understand and authorize the access, use and disclosure of my information as stated in this form. I know that being in this study is voluntary. I choose to be in this study: I will get a signed and dated copy of this consent form.

Name of Subject:____
Signature:____ Date:______________

Print Name:____

Consent form explained by:_________________________________ Date:______________
Print Name: ____
